# The Effects of Tacrolimus on Tissue-Specific, Protein-Level Inflammatory Networks in Vascularized Composite Allotransplantation

**DOI:** 10.3389/fimmu.2021.591154

**Published:** 2021-05-04

**Authors:** Ali Mubin Aral, Ruben Zamora, Derek Barclay, Jinling Yin, Fayten El-Dehaibi, Vasil E. Erbas, Liwei Dong, Zhaoxiang Zhang, Huseyin Sahin, Vijay S. Gorantla, Yoram Vodovotz

**Affiliations:** ^1^ Department of Surgery, University of Pittsburgh, Pittsburgh, PA, United States; ^2^ Center for Inflammation and Regenerative Modeling, McGowan Institute for Regenerative Medicine, University of Pittsburgh, Pittsburgh, PA, United States; ^3^ Department of Plastic, Reconstructive and Aesthetic Surgery, Medicalpark Gaziantep Hospital, Gaziantep, Turkey; ^4^ Plastic and Aesthetic Surgery Department, XiJing Hospital, Xi’an, China; ^5^ Private Cagsu Hospital, Duzce, Turkey; ^6^ Department of Surgery, Wake Forest Institute for Regenerative Medicine, Wake Forest Baptist Medical Center, Winston Salem, NC, United States

**Keywords:** vascularized composite allotransplantation, inflammation, systems biology, network analysis, immunosuppression, acute rejection

## Abstract

Systems-level insights into inflammatory events after vascularized composite allotransplantation (VCA) are critical to the success of immunomodulatory strategies of these complex procedures. To date, the effects of tacrolimus (TAC) immunosuppression on inflammatory networks in VCA, such as in acute rejection (AR), have not been investigated. We used a systems biology approach to elucidate the effects of tacrolimus on dynamic networks and principal drivers of systemic inflammation in the context of dynamic tissue-specific immune responses following VCA. Lewis (LEW) rat recipients received orthotopic hind limb VCA from fully major histocompatibility complex-mismatched Brown Norway (BN) donors or matched LEW donors. Group 1 (syngeneic controls) received LEW limbs without TAC, and Group 2 (treatment group) received BN limbs with TAC. Time-dependent changes in 27 inflammatory mediators were analyzed in skin, muscle, and peripheral blood using Principal Component Analysis (PCA), Dynamic Bayesian Network (DyBN) inference, and Dynamic Network Analysis (DyNA) to define principal characteristics, central nodes, and putative feedback structures of systemic inflammation. Analyses were repeated on skin + muscle data to construct a “Virtual VCA”, and in skin + muscle + peripheral blood data to construct a “Virtual Animal.” PCA, DyBN, and DyNA results from individual tissues suggested important roles for leptin, VEGF, various chemokines, the NLRP3 inflammasome (IL-1β, IL-18), and IL-6 after TAC treatment. The chemokines MCP-1, MIP-1α; and IP-10 were associated with AR in controls. Statistical analysis suggested that 24/27 inflammatory mediators were altered significantly between control and TAC-treated rats in peripheral blood, skin, and/or muscle over time. “Virtual VCA” and “Virtual Animal” analyses implicated the skin as a key control point of dynamic inflammatory networks, whose connectivity/complexity over time exhibited a U-shaped trajectory and was mirrored in the systemic circulation. Our study defines the effects of TAC on complex spatiotemporal evolution of dynamic inflammation networks in VCA. We also demonstrate the potential utility of computational analyses to elucidate nonlinear, cross-tissue interactions. These approaches may help define precision medicine approaches to better personalize TAC immunosuppression in VCA recipients.

## Introduction

The potential for vascularized composite allotransplantation (VCA) as a reconstructive or restorative option has been established in over 200 procedures performed worldwide, including 145 upper extremity (hand), 42 face, and 26 uterus transplants (with over 10 live births). Nevertheless, the risks of lifelong, high-dose or multi-drug systemic immunosuppression remain the key challenge limiting life enhancing benefits of VCA (1–3).

Tacrolimus (TAC; FK506, Prograf ^®^), is an FDA-approved immunosuppressant which is the keystone agent in VCA protocols (4, 5). Although TAC is mostly effective in controlling acute rejection (AR), it does not prevent chronic rejection (CR). Multiple hand transplant (6, 7) and face transplant recipients have succumbed to CR (8, 9). The main limitation of TAC is its narrow therapeutic range, which underlies its toxic side effects (10–12). Excessive immunosuppression with TAC can lead to nephrotoxicity, malignancy, or opportunistic infection); inadequate immunosuppression can increase risks of AR or CR.

VCA tissues are primary triggers and targets of the host adaptive and effector immune responses, with draining lymph nodes serving as the primary sites of allorecognition (13). Conventional/standard immunosuppression regimens have prolonged VCA graft survival; however, in most cases, standard baseline immunosuppression (such as TAC) fails to fully prevent long-term skin rejection/deterioration. This is because skin in VCA is highly immunogenic due to unique cellular, cytokine, and chemokine networks (14, 15).

Notwithstanding the antigenicity of the skin, inflammation is the primary determinant of rejection post-VCA (16). Computational modeling suggests that this could be caused by cytokine- or chemokine-mediated immune events such as ischemia-reperfusion injury (IRI), surgical inflammation (SI), and AR after transplantation in general, including VCA (17). Though properly-regulated inflammation allows for timely recognition and repair of traumatic injury and subsequent VCA, insufficient (18) or self-sustaining (19) inflammation can lead to long-lasting immune dysregulation (20). This prolonged immune dysregulation and ongoing tissue damage, can, in turn, predispose to transplant rejection in various contexts (21, 22), including VCA (16, 23, 24).

The success of systemic immunosuppressive therapies or other immunomodulatory strategies is hampered by our lack of understanding of the key dynamic networks and principal drivers of such local/systemic inflammation after VCA. We and others have previously utilized systems and computational biology approaches to help decipher the complexity of inflammation in the context of trauma, sepsis, and wound healing, under the rubric of translational systems biology of inflammation (25, 26). Here, we implement a unique translational systems biology approach, extending initial work characterizing dynamic inflammatory changes in experimental VCA (27, 28). Our overall objective was to model protein-level inflammation networks after limb transplantation, focusing on the early and ongoing local (skin, muscle) and systemic (peripheral blood) processes and their cross-interactions, as well as investigating the effects of TAC on these complex dynamics.

## Materials and Methods

This study utilized the following workflow, which is detailed below. Briefly, rats were subjected to syngeneic or allogeneic hind limb transplantation with TAC, followed by analysis of multiple inflammatory mediators at various time points and integration of the data *via* various machine learning methods.

### Rat Hind Limb Transplantation Model

The study was approved by the Institutional Animal Care and Use Committee (IACUC) of the University of Pittsburgh and by the Department of Defense Animal Care and Utilization Review Office (ACURO). Full MHC mismatched, male Lewis (LEW) and Brown Norway (BN) rats (Charles River Laboratories, OH), 10-12 weeks age and weighing 300-320 grams, were used in this study. Rats were anesthetized using isoflurane. A circumferential incision was made in a LEW or BN rat donor, at the level of inguinal ligament to expose femoral artery and vein. Epigastric vessels were ligated, and dissected femoral vessels were transected at the level of inguinal ligament. The two limbs were amputated at the level of middle femur. The recipient LEW rat was prepared similarly. Bone fixation was performed with 18-gauge needle. Muscle groups were repaired with 4/0 Vicryl (Ethicon Inc., Somerville, NJ). Femoral artery and vein anastomosis were done with 10-0 Nylon stitches (Aros Surgical Inc, CA). Skin was closed with 4-0 Nylon suture (Aros Surgical Inc, CA) (29).

### Experimental Design

Due to limitations associated with the animal protocol, a maximum of 3-4 samples were obtained from any given experimental animal, for a total of 7 time-points across multiple animals (d0-d20) as shown in detail in the **Supplementary Material**. Both the rats and time points were randomized. Hierarchical clustering analysis of all the data broken down by rats showed no clear clustering based on rats (as shown in **Supplementary Figure 1** for the plasma data), suggesting the absence of any batch effects. **Group 1** (syngeneic controls, n=84 samples across all time-points): LEW recipients received full MHC-matched LEW hindlimbs without TAC. **Group 2** (treatment group, n=84 samples across all time-points): LEW recipients received full MHC-mismatched BN limbs with TAC (1 mg/kg/day, a dose used in prior studies utilizing this animal model (30)) administered intraperitoneally until postoperative day (POD) 20 followed by drug withdrawal. **Group 3** (rejection controls, n=8): LEW recipients received full MHC-mismatched BN hindlimbs without TAC. The primary comparison in this study was between Groups 1 and 2, with Group 3 serving as a comparator to demonstrate the timeline and intensity of AR in the absence of immunosuppression.

### Analysis of Inflammatory Mediators

Skin and muscle tissue samples were collected from hind limbs at 0, 3, 5, 7, 9, 11, and 20 days in RNAlater solution (Sigma-Aldrich, St. Louis, MO) in addition to peripheral blood samples, and all were stored at -80°C until analysis. Total protein isolation and determination were carried out using the BCA protein assay kit from Pierce (Rockford, IL) with bovine serum albumin as standard as previously described (31). Rat inflammatory mediators were measured using a Luminex™ MagPix apparatus (Luminex, Austin, TX) and antibody kit (EMD Millipore Kit, Billerica, MA) as per manufacturer’ s specifications. The antibody bead kit included: Eotaxin (CCL11), Granulocyte Colony-Stimulating Factor (G-CSF), Granulocyte Macrophage Colony-Stimulating factor (GM-CSF), Keratinocyte-derived Cytokine (Gro-α/KC/CXCL1), Interferon-γ (IFN-γ), Interleukin (IL)- 1α, IL-1β, IL-2, IL-4, IL-5, IL-6, IL-10, IL-12p70, IL-13, IL-17A, IFN-γ– inducible Protein 10 (IP-10/CXCL10), Leptin, Monocyte Chemoattractant Protein (MCP-1/CCL2), Macrophage Inflammatory Protein-1α (MIP-1α/CCL3), MIP-2/Gro-β, Tumor Necrosis Factor-α (TNF-α), Regulated on Activation, Normal T cell Expressed and Secreted (RANTES/CCL5), and Vascular Endothelial Growth Factor (VEGF). The final mediator concentrations are expressed in pg/mg protein (skin and muscle) or pg/ml (peripheral blood) as indicated. Experimental data are presented as mean ± SEM.

### Histopathology

Rat limb allografts were assessed daily for evidence of rejection by inspection. Skin rejection was classified per appearance according to a 5‐point Banff grading scale: grade 0—no signs of rejection; grade I—erythema; grade II—erythema and edema; grade III—epidermolysis; grade IV—mummification and necrosis (32, 33). Skin and muscle tissue samples (4 mm punch) were collected from hind limbs at 0, 3, 5, 7, 9, 11, 20, 23, 25, 27 and 31 days (with time points past 20 days being obtained only in order to define the degree of protection from AR afforded by TAC histologically, but not used in computational analyses). Animals were sacrificed at specific time points until the 31-day study end point or upon Grade III Banff AR – whichever occurred earlier. Specimens of allograft skin and muscle were formalin‐fixed and paraffin‐embedded or snap‐frozen in liquid nitrogen. Sections were H&E stained and evaluated for lymphocytic infiltration, dermal/epidermal interphase reaction and necrosis by a pathologist in a blinded fashion.

### Statistical and Computational Analyses

Time-dependent changes of inflammatory mediator levels were assessed for significance using One-Way Analysis of Variance (ANOVA). Comparison between experimental groups (syngeneic-control vs. allogeneic-TAC) was carried out by Two-Way ANOVA followed by Holm-Sidak *post hoc* test (significance set at P<0.05) using Sigma Plot (Systat Software, San Jose, CA) as indicated.

Dynamic Bayesian Network (DyBN) inference (34) is a technique to define the most likely single network structure that best characterizes the dynamic interactions among systemic inflammatory mediators across all time points, in the process suggesting likely feedback structures that define central nodes. The networks may also suggest possible mechanisms by which the progression of the inflammatory response differs within a given experimental subgroup. In this analysis, time courses of unprocessed inflammatory mediator measurements were used as input for a DyBN inference algorithm (34), implemented in MATLAB^®^ essentially as described previously (34) and modified by our group for the study of systemic acute inflammation (35–37). In brief, given time-series data, DyBN analysis provides a way of inferring causal relationships among variables (e.g. inflammatory mediators) based on probabilistic measure. Unlike standard correlative approaches, DyBNs consider the joint distribution of the entire dataset when making inferences about the dependencies between variables or nodes in the network. The values of each node are assumed to be distributed according to a chosen model (e.g. Gaussian) and the relationships among nodes are defined by the structure of the directed network and the corresponding conditional probability distributions of the interacting nodes. Network structure is inferred by a sampling technique that iteratively proposes candidate structures and evaluates them based on how well they fit the observed data using a specified scoring criterion, until reaching convergence on a network structure with the highest score. The algorithm uses an inhomogeneous dynamic changepoint model, with a Bayesian Gaussian with score equivalence (BGe) scoring criterion. The output of the aforementioned algorithm is a final graph structure indicating the interactions. Central/high-feedback nodes are those that exhibit self-feedback in addition to being connected to other nodes. Feedback loops can be important in systemic inflammation for control/resolution.

Dynamic Network Analysis (DyNA) (38) was aimed to define the central inflammatory network nodes as a function of both time and treatment in a granular fashion over time intervals; and unlike DyBN, DyNA is deterministic and cannot represent feedback structures but is useful for defining hub nodes at distinct time intervals. Rats underwent hind-limb VCA along hind-limb VCA along with syngeneic and allogeneic groups as described and peripheral blood samples were obtained at postoperative 0, 3, 5, 7, 9, 11, and 20 days. Using inflammatory mediator measurements of three time points for each experimental group, networks were created over two consecutive periods (day 3-5, day 5-7, day 9-11, and day 11-20) using MATLAB^®^ software; data were not binned. Connections ([edges], or number of trajectories of inflammatory mediators that move in parallel [black edges = positive correlations] or in an anti-parallel [red edges = negative correlations] fashion) were created if the Pearson correlation coefficient between any two nodes (inflammatory mediators) at the same time interval was greater or equal to a threshold of an absolute value of 0.7, indicating a strong correlation. The network connectivity/complexity for each time interval was calculated using the following formula: Sum (N_1_ + N_2_ +… + Nn)/(n−1), where N represents the number of connections for each mediator, and n is the total number of mediators analyzed.

Principal component analysis (PCA) (39) was carried out to identify the inflammatory mediators that contributed the most to the overall variance of the response in peripheral blood of rats that underwent hind-limb VCA along with syngeneic and allogeneic groups (0-20 days) using normalized data, as described (38). To represent the 3D-PCA results for the first three main components, we employed MetaboAnalyst, a web-based tool suite developed for comprehensive metabolomic data analysis that also supports a wide array of functions for statistical, functional, as well as data visualization tasks (https://www.metaboanalyst.ca). To allow for comparison of experimental groups and tissue sources with similar variance, we calculated the contribution of each mediator to the overall variance (99.99%) and then selected those (highlighted in colored boxes) contributing to the top 25% variance of the inflammatory response in each tissue in each group as reported previously (40), using MATLAB^®^ software (The MathWorks, Inc., Natick, MA). Data were not binned.

## Results

### Histopathology

**Figure 1:**
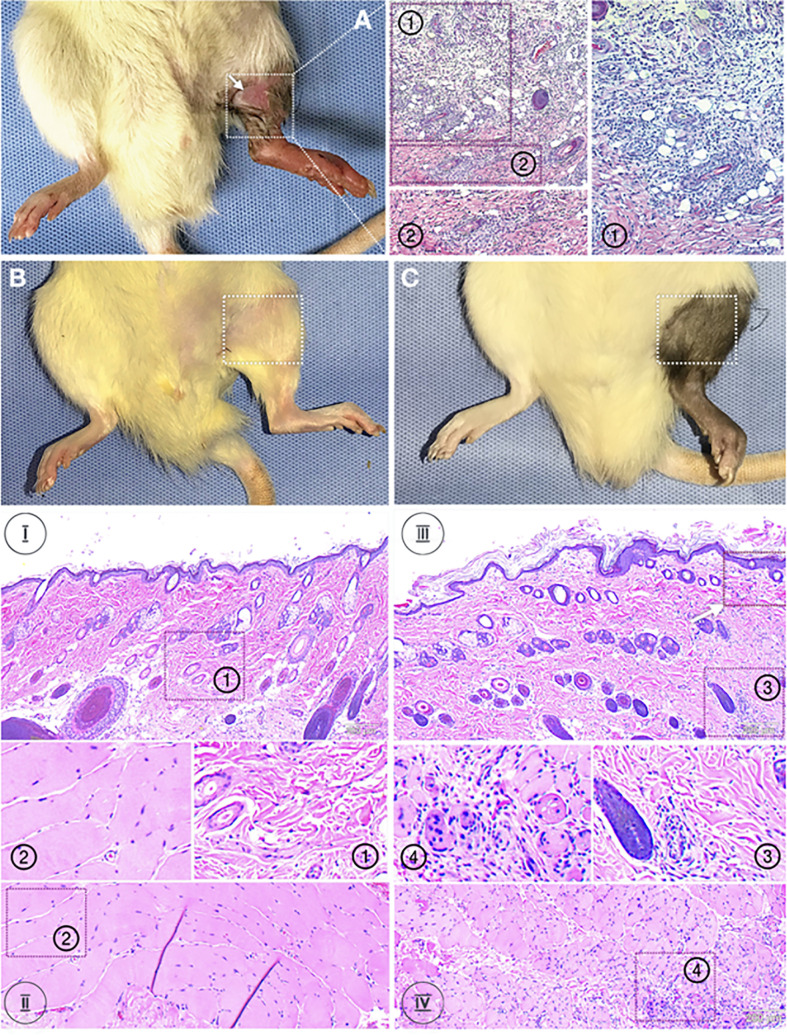
Histology of skin and muscle samples. Animals were randomized into two experimental groups: Group 1 (n=8, syngeneic controls; LEW recipients receiving MHC-matched LEW limbs without TAC. Group 2 (n=8, treatment group) LEW recipients received full MHC-mismatched BN limbs with TAC administered until postoperative day 20 followed by drug withdrawal. Representative animals from each group were euthanized and VCA tissues analyzed for inflammatory and other pathology. Panel **(A)**: a skin (1) and muscle (2) biopsy from an allogeneic transplant without TAC. These transplants undergo complete rejection by 7 +/- 2 days after surgery with epidermolysis and hair loss (white arrow), and necrosis of epidermis with severe, dense inflammation in upper and deep dermis and muscle, respectively (*Grade II to IV Banff AR*) (1 and 2). Panel **(B)**: Group 1 (syngeneic transplants). Skin and muscle biopsies at POD 31 revealed no inflammatory infiltrates in the epidermis, dermis, or muscle. Panel **(C)**: Group 2 (allogeneic transplants with TAC) were followed until POD 31 following withdrawal of TAC at POD 20. Despite absence of clinical signs of rejection, the epidermis showed mild perivascular infiltration without epidermal dyskeratosis or apoptosis (white arrow). The deep dermis showed mild to moderate peri-adnexal lymphocyte infiltration with involvement of hair follicles as well as muscle (*Grade I to II Banff AR*).

Skin and muscle biopsies at POD 31 from Group 1 (syngeneic transplants) (**Figure 1B**) revealed no inflammatory infiltrates in epidermis or dermis [**Figure 1B** (1)] as well as in the muscle [**Figure 1B** (2)]. Animals in Group 2 (allogeneic transplants with TAC) were followed until POD 31 following withdrawal of TAC at POD 20 (**Figure 1C**). Despite absence of clinical signs of rejection, the epidermis showed mild perivascular infiltration without epidermal dyskeratosis or apoptosis (**Figure 1C** (3), white arrow). The deep dermis showed mild to moderate peri-adnexal lymphocyte infiltration with involvement of hair follicles (**Figure 1C** (3) as well as muscle (**Figure 1C** (4); *Grade I to II Banff AR* [indicated as “I” and “II”]). **Figure 1A** (Group 3) shown here for comparison only is a skin (1) and muscle (2) biopsy from an allogeneic transplant without TAC, maintained only out to 11 days due to animal care regulations. These transplants in Group 3 undergo complete rejection by 7 +/- 2 days after surgery with epidermolysis and hair loss (white arrow), and necrosis of epidermis with severe, dense inflammation in upper and deep dermis and muscle, respectively (*Grade II to IV Banff AR*) [**Figure 1A** (1 and 2)].

### Inflammatory Mediators in the Skin, Muscle, and Peripheral Blood After VCA Exhibit Differential Dynamic Trajectories

We next sought to define dynamic changes in inflammatory mediators in the skin, muscle, and systemic circulation in Group 2 (representing the clinical scenario) versus Group 1 (syngeneic) controls. One-Way ANOVA of time-courses showed that animals receiving TAC had the greatest temporal change in inflammatory mediators in peripheral blood (17/27 [63%]), followed by skin (14/27 [52%]) and muscle (11/27 [41%]) (**Supplementary Table 1**). The muscle was the tissue with the greatest degree of temporal inflammatory changes in the syngeneic control group (13/27 mediators [52%]) with fewer changes in skin and peripheral blood (7/27 mediators [44%] each) (**Supplementary Table 1**). Furthermore, comparison by Two-Way ANOVA of Groups 2 vs. 1 revealed significant differences in multiple inflammatory mediators, as shown in **Supplementary Figure 2**.

### Dynamic Inflammatory Networks After VCA in Skin, Muscle and Peripheral Blood Are Governed by Distinct Central Nodes

**Figure 2:**
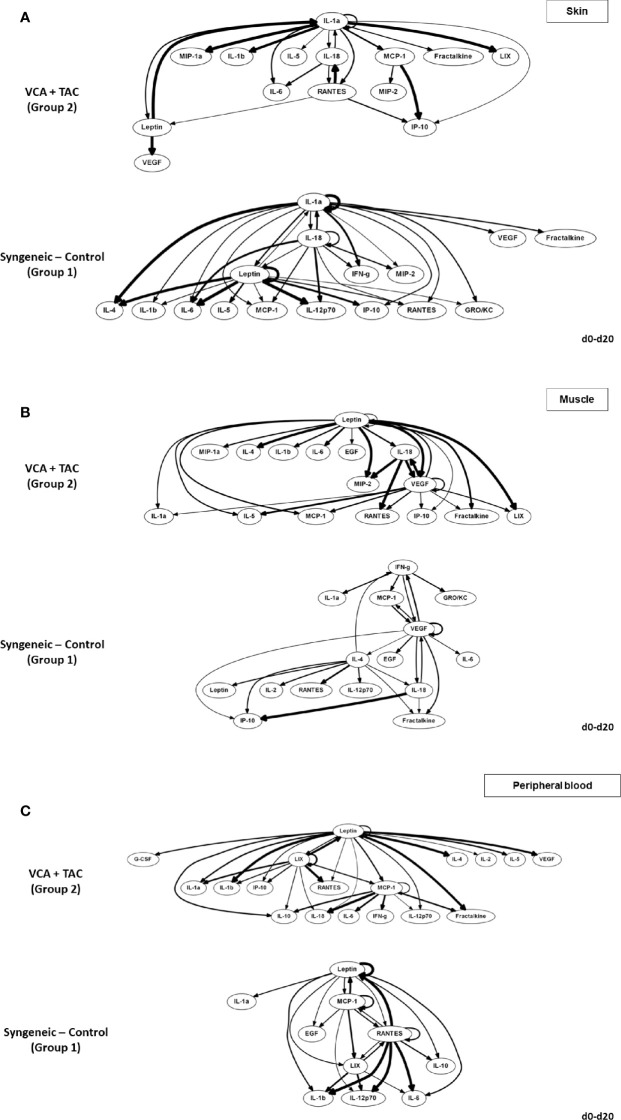
Differential DyBN network patterns in rats undergoing VCA + TAC vs. Syngeneic Control. LEW rat recipients received full MHC-mismatched BN limbs with TAC (1 mg/kg/day, i.p.) until postoperative day 20 followed by drug withdrawal as described in *Materials and Methods*. LEW rat recipients that received MHC-matched LEW limbs without TAC served as control. Peripheral blood, skin and muscle tissue samples were collected at 0, 3, 5, 7, 9, 11, and 20 days and assayed for 27 inflammatory mediators using the rat multiplex Luminex™ assay followed by DyBN analysis as described in *Materials and Methods*. Panels **(A–C)** show the inflammatory networks for skin, muscle, and peripheral blood, respectively. This analysis suggested the presence of distinct central nodes in the skin, muscle, and peripheral circulation of animals undergoing VCA + TAC as compared to syngeneic transplant controls.

We used DyBN inference to highlight putative feedbacks among variables and to identify inflammatory mediators that might act as central controllers of VCA-induced inflammation. We considered as central those nodes that exhibit self-feedback and connect to other nodes (i.e., these nodes were high-feedback nodes). Based on this definition, IL-1α was a central node in skin samples from Group 2 animals receiving TAC, upstream of IL-18, RANTES (which all exhibited mutual cross-interactions) as well as multiple additional downstream mediators (**Figure 2A**, upper panel). In contrast, IL-1α, IL-18, and leptin appeared as central nodes in the skin of Group 1 (syngeneic) animals (**Figure 2A**, lower panel). This network pattern was interconnected with multiple additional mediators, suggesting a central inflammatory role for pathways involving leptin, pyroptosis, and the NLRP3 inflammasome. Leptin and VEGF were central nodes in the muscle of Group 2, interconnected with IL-1α and IL-18 as well as multiple other inflammatory mediators (**Figure 2B**, upper panel). In comparison, VEGF was the sole central node in the muscle of Group 1 animals, with two-way connections to IFN-γ and MCP-1 along with various connections to multiple inflammatory mediators (**Figure 2B**, lower panel). These results suggest the possible role of tissue ischemia (VEGF) along with the leptin pathway.

The systemic (peripheral blood) inflammatory response in the presence of TAC (Group 2) was inferred to involve leptin, LIX, and MCP-1 as central nodes, upstream of a complex and highly interconnected set of inflammatory mediators (**Figure 2C**, upper panel). Group 1 rats exhibited a systemic inflammatory response that was less complex, with leptin, MCP-1, and RANTES as central nodes (**Figure 2C**, lower panel). These results suggest a relatively similar core systemic inflammatory response in Group 1 and 2, albeit with a more complex overall response with TAC treatment.

### Inflammatory Networks in Skin, Muscle and Peripheral Blood Are Characterized by Unique Time-Dependent Evolution After VCA

We used DyNA for the granular visualization of the temporal evolution of inflammatory networks after VCA. Dynamic Networks were analyzed at POD 3-5, POD 5-7, POD 7-9, POD 9-11, and POD 11-20. In this analysis, inflammatory mediators are depicted as network nodes (red circles in **Supplementary Figure 3**), and the statistical correlation among nodes is depicted as edges (black or red lines interconnecting any two nodes, with black lines representing a positive correlation and red lines representing a negative correlation).

**Figure 3:**
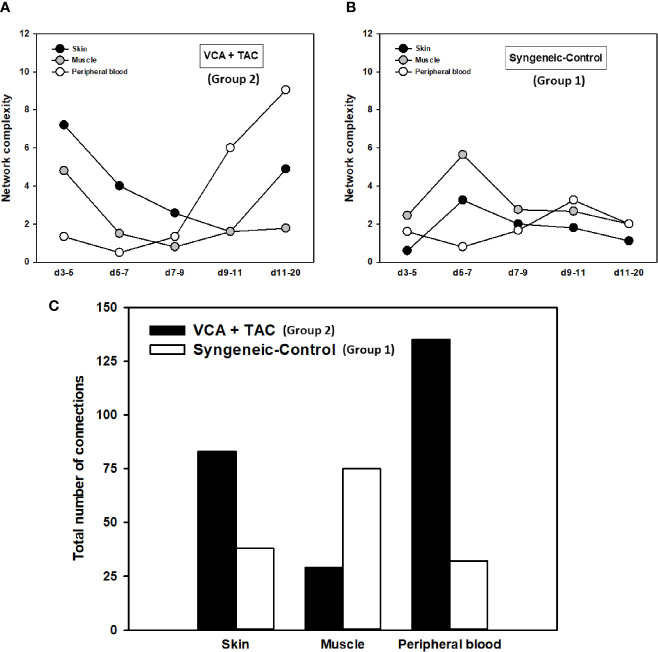
Dynamic inflammatory networks in skin, muscle and peripheral blood are characterized by unique time-dependent evolution after VCA. LEW rat recipients received full MHC-mismatched BN limbs with TAC (1 mg/kg/day, i.p.) until postoperative day 20 followed by drug withdrawal as described in *Materials and Methods*. LEW rat recipients that received MHC-matched LEW limbs without TAC served as control. Peripheral blood, skin and muscle tissue samples were collected at 0, 3, 5, 7, 9, 11, and 20 days and assayed for 27 inflammatory mediators using the rat multiplex Luminex™ assay followed by DyNA performed during five time-intervals as described in *Materials and Methods.* Figure shows the network complexity (stringency level 0.7) in skin, muscle, and peripheral blood in VCA+TAC **(A)** vs. Syngeneic-Control **(B)** and total number of connections **(C)** calculated as described in *Materials and Methods.* This analysis suggested the presence of distinct dynamic networks and network connectivity/complexity patterns in the skin, muscle, and peripheral circulation of animals undergoing VCA + TAC as compared to syngeneic transplant controls.

A key metric of these networks, namely network connectivity/complexity quantifies these qualitative impressions. Analysis of the dynamic connectivity/complexity of the inflammatory networks suggested that networks under TAC treatment (Group 2) evolve quite differently over time in skin, muscle, and peripheral blood as compared to without TAC (Group 1) (**Figure 3**). The general dynamic pattern in Group 2 suggested a high initial network connectivity/complexity in skin and muscle that was reduced by POD 7-9, but which then rose through POD 11-20 (**Figure 3A**). In contrast, inflammatory network connectivity/complexity in all tissues of syngeneic transplant (Group 1) animals was trending towards resolution over the full time period studied (**Figure 3B**). An analysis of the total number of network connections over time suggested a greater network connectivity in peripheral blood and skin of TAC treated vs. syngeneic transplant; however, the opposite pattern was observed in the muscle (**Figure 3C**). It was also interesting to note that the highest network connectivity in TAC-treated animals was found in the peripheral blood, while the highest network connectivity in syngeneic transplants was observed in the muscle (**Figure 3C**). The results are summarized in **Table 1**.

**Table 1:** Summary of the main differences between Syngeneic (Control) and Treatment (VCA+TAC) groups.

Experimental Condition	Statistically altered mediators	Principal Drivers (PCA)	Dynamic Networks (DyNA)	Central Nodes (DyBN)
***Group 1***	**Skin:**	**Skin:**		**Skin:**
Syngeneic Control	Leptin, MIP-1α, IL-1β, IL-2, IL-18, MCP-1	MIP-2, IL-6, LIX	High network complexity resolving over POD 11-20	IL-1α, IL-18, leptin
LEW-LEW transplants with no TAC				
	**Muscle:**	**Muscle:**	Muscle had highest network connectivity	**Muscle:**
	IL-1α, IL-4, IL-2, EGF, IL-10, Il-12p70, IL-5, IL-18, MCP-1, IP-10, VEGF, Fractalkine, RANTES	MIP-1α, GRO/KC, IL-6, Leptin		VEGF
	**Blood:**	**Blood:**		**Blood:**
	MIP-1α, IL-1β, EGF, IL-10, MCP-1, MIP-2, RANTES	IFN-γ, EGF, GRO/KC, IL-10		Leptin, MCP-1, RANTES
***Group 2***	**Skin:**	**Skin:**		**Skin:**
Treatment Group	Eotaxin, MIP-1α, IL-4, IL-6, IL-13, IL-12p70, IL-17A, IL-18, MCP-1, IP-10, VEGF, Fractalkine, TNF- α, RANTES	IL-12p70, IL-13, LIX, MIP-2, IL-4	Initially high network complexity decreasing through POD 7-9; increasing through POD 11-20	IL-1α
LEW-BN transplants with TAC 1mg/kg until POD20				
	**Muscle:**	**Muscle:**	Peripheral blood had highest network connectivity	**Muscle:**
	Eotaxin, Leptin, IL-4, IL-1β, IL-13, IL-18, MCP-1, IP-10, VEGF, LIX, RANTES	IL-13, IL-1β, LIX, IL-17A,		Leptin, VEGF
	**Blood:**	**Blood:**		**Blood:**
	G-CSF, Eotaxin, Leptin, MIP-1α, IL-2, IL-6, IL-13, IL-12p70, IL-17A, IL-18, MCP-1, IP-10, VEGF, LIX, MIP-2, TNFα, RANTES	IL-6, IL-17A, IFN-γ, MIP-2		Leptin, LIX and MCP-1

### Principal Component Analysis Suggests a Differential Inflammatory Response After VCA

**Figure 4:**
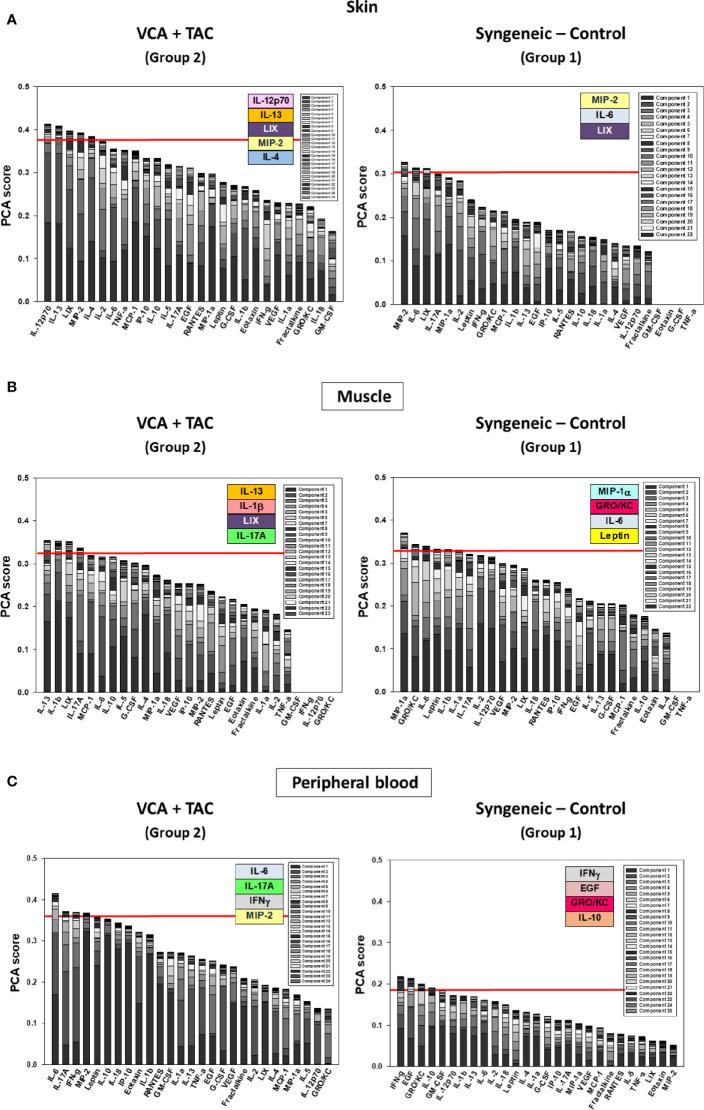
Principal Component Analysis (PCA) suggests differential inflammatory characteristics after VCA + TAC vs. Syngeneic-Control. LEW rat recipients received full MHC-mismatched BN limbs with TAC (1 mg/kg/day, i.p.) until postoperative day 20 followed by drug withdrawal as described in *Materials and Methods*. LEW rat recipients that received MHC-matched LEW limbs without TAC served as control. Peripheral blood, skin and muscle tissue samples were collected at 0, 3, 5, 7, 9, 11, and 20 days and assayed for 27 inflammatory mediators using the rat multiplex Luminex™ assay followed by PCA performed as described in *Materials and Methods.* The number of mediators contributing to the top 25% variance of the inflammatory response (shown above the red line) are highlighted in colored boxes in VCA + TAC vs. Syngeneic-Control in skin **(A)**, muscle **(**Panel **B)** and peripheral blood **(**Panel **C)** as indicated.

We used PCA to define the key variables contributing to the variance in a given dataset, especially a dataset which represent a dynamic process. An analysis using the first three principal components accounted for widely differing degrees of variance depending on the tissue assessed and suggested that the two experimental groups could not be separated (**Supplementary Figure 4**). Accordingly, we sought to account for a uniform degree of variance (99.9%) and then compare the top 25% of the mediators in each tissue in each group as reported previously (40) Based on this analysis, the skin (**Figure 4A**), muscle (**Figure 4B**), and peripheral blood (**Figure 4C**) in TAC-treated animals (Group 2) were inferred to involve a differential set of mediators in each tissue as compared to those observed after syngeneic transplants (Group 1). The results are summarized in **Table 1**.

### *In Silico* VCA: Merging Skin and Muscle Data on Computational Analyses Reveals Complex, Cross-Tissue Crosstalk After VCA

A key aspect of VCA is the multi-tissue nature of the transplant. Previous studies (13, 28), as well our current findings thus far, have examined the inflammatory response in individual components (skin and muscle). However, it is likely that cross-tissue interactions mediate the inflammatory and immune response in the setting of VCA (41). However, it is difficult, if not impossible, to define experimentally and directly the tissue-specific inflammatory contributions of skin and muscle. We therefore next hypothesized that we could integrate the skin and muscle data on inflammatory cytokines using DyBN, DyNA, and PCA to define such cross-tissue interactions (in essence defining an “*in silico* VCA”).

**Figure 5:**
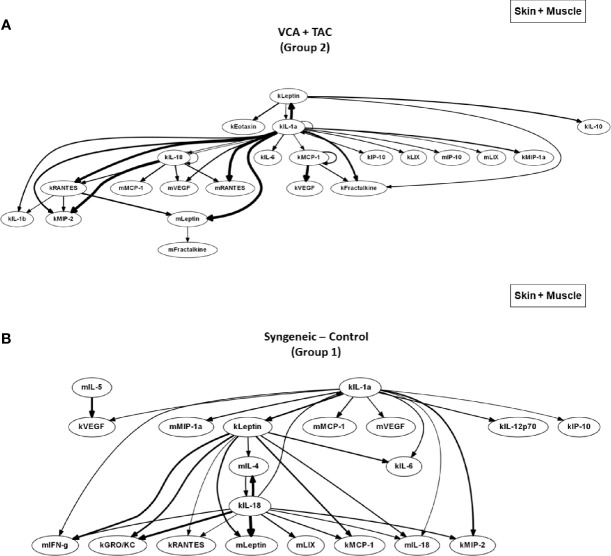
DyBN network patterns in rats undergoing VCA +TAC vs. Syngeneic Control. LEW rat recipients received full MHC-mismatched BN limbs with TAC (1 mg/kg/day, i.p.) until postoperative day 20 followed by drug withdrawal as described in *Materials and Methods*. LEW rat recipients that received MHC-matched LEW limbs without TAC served as control. Skin and muscle tissue samples were collected at 0, 3, 5, 7, 9, 11, and 20 days and assayed for 27 inflammatory mediators using the rat multiplex Luminex™ assay followed by DyBN analysis (skin and muscle data combined) as described in *Materials and Methods*. Panels **(A, B)** show the inflammatory networks for VCA + TAC and Syngeneic-Control, respectively.

**Figure 6:**
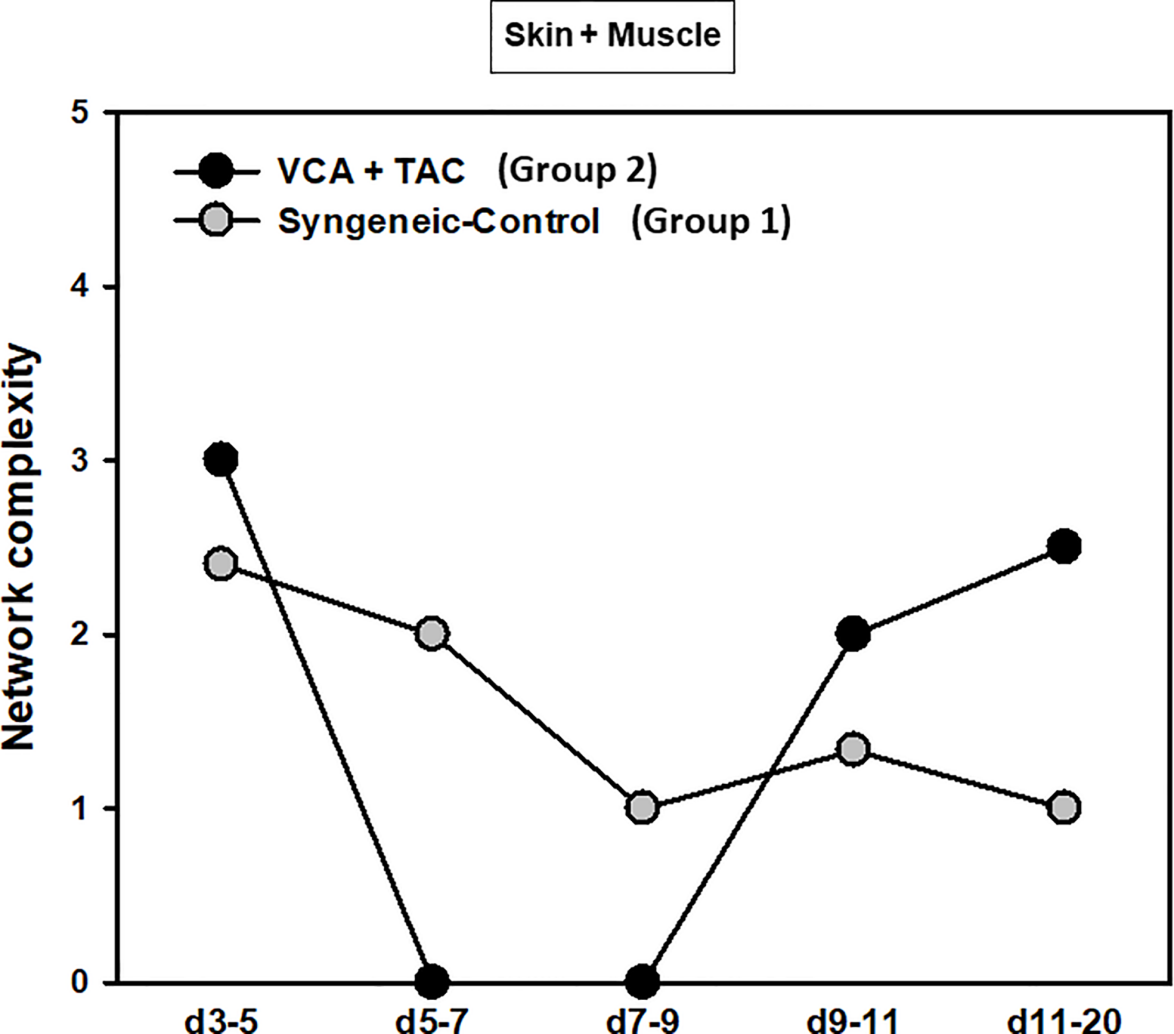
Dynamic inflammatory networks in skin and muscle are characterized by differential time-dependent evolution after VCA. LEW rat recipients received full MHC-mismatched BN limbs with TAC (1 mg/kg/day, i.p.) until postoperative day 20 followed by drug withdrawal as described in *Materials and Methods*. LEW rat recipients that received MHC-matched LEW limbs without TAC served as control. Skin and muscle tissue samples were collected at 0, 3, 5, 7, 9, 11, and 20 days and assayed for 27 inflammatory mediators using the rat multiplex Luminex™ assay followed by DyNA performed during five time-intervals as described in *Materials and Methods*. Figure shows the network complexity (stringency level 0.7) in skin and muscle (combined) in VCA+TAC vs. Syngeneic-Control calculated as described in *Materials and Methods*.

**Figure 7:**
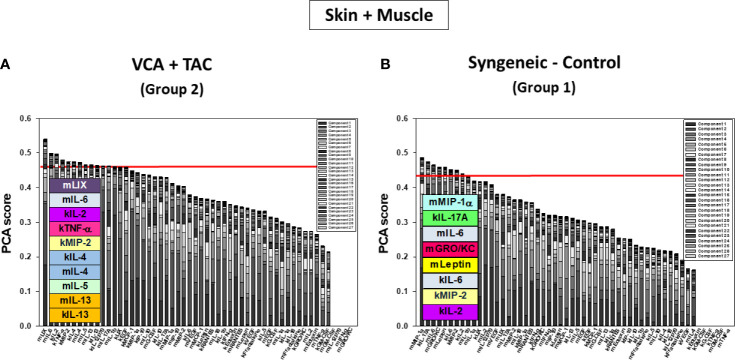
Principal Component Analysis (PCA) suggests differential inflammatory characteristics after VCA + TAC vs. Syngeneic-Control. LEW rat recipients received full MHC-mismatched BN limbs with TAC (1 mg/kg/day, i.p.) until postoperative day 20 followed by drug withdrawal as described in *Materials and Methods*. LEW rat recipients that received MHC-matched LEW limbs without TAC served as control. Skin and muscle tissue samples were collected at 0, 3, 5, 7, 9, 11, and 20 days and assayed for 27 inflammatory mediators using the rat multiplex Luminex™ assay followed by PCA performed as described in *Materials and Methods.* The number of mediators contributing to the top 25% variance of the inflammatory response (shown above the red line) are highlighted in colored boxes in skin and muscle (combined) in VCA + TAC **(A)** vs. Syngeneic-Control **(B)** as indicated.

DyBN of combined skin and muscle data supported the notion that there is complex, cross-tissue crosstalk in both TAC treated as well as syngeneic recipients. With TAC treatment, skin MCP-1, IL-1α, and IL-18, showed multiple connections to inflammatory mediators in skin and muscle combined (**Figure 5A**). Leptin, while not a central node, exhibited two-way feedback with IL-1α (**Figure 5A**). In syngeneic transplants, there were no discernible central mediators, though muscle IL-4 and skin IL-18 exhibited two-way interactions (**Figure 5B**). DyNA of the skin + muscle data suggested that, as inferred from the single-tissue data, inflammatory networks in VCA + TAC animals exhibited dropped to zero network connectivity/complexity between POD 5-9, and then increased to levels higher than those of syngeneic transplant animals through day 20. In contrast, inflammatory networks in syngeneic transplant animals trended towards resolution of inflammation over time (**Figure 6**). Detailed inflammatory networks are depicted in **Supplementary Figure 5**. Finally, PCA suggested a fairly similar overall degree of inflammatory activation with TAC treatment (Group 2) and without it (Group 1), but with different principal characteristics (**Figure 7**).

### *In Silico* Animal: Integrating Whole-Body Level (Skin + Muscle + Peripheral Blood) Data Suggests a Role for Inflammasome Interactions After VCA+TAC

The expression of CYP3A5 genes that encode monooxygenases important for TAC metabolism *in vivo* is negligible in VCA components such as the skin, skeletal muscle, or adipose tissue as compared to the liver or intestine, which are the major sites of TAC metabolism (42). Therefore, differences in pharmacokinetics and pharmacodynamics of TAC across different tissues, combined with differential partitioning of TAC in peripheral blood (RBC or lymphocytes) versus tissues may underlie the complex interplay of cytokines and chemokines that drive immune rejection in VCA (43).We therefore integrated the skin and muscle data with data on the systemic (peripheral blood) response of inflammatory mediators using DyBN, DyNA, and PCA (in essence defining an “*in silico* animal”), with the goal of inferring local and systemic cross-talk and potentially gaining insights into these complex effects of TAC.

**Figure 8:**
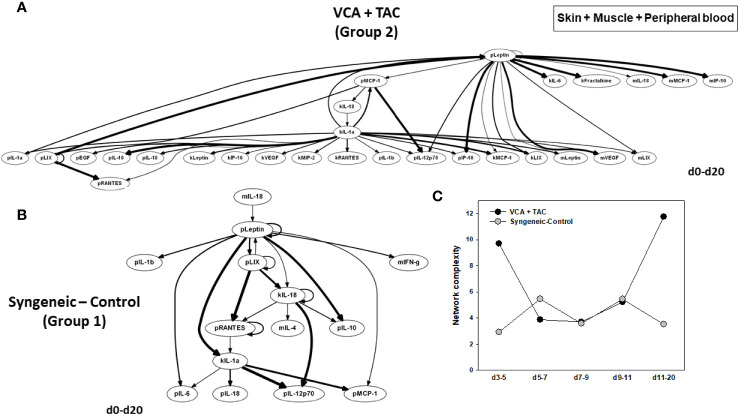
Differential inflammatory network patterns in rats undergoing VCA +TAC vs. Syngeneic Control. LEW rat recipients received full MHC-mismatched BN limbs with TAC (1 mg/kg/day, i.p.) until postoperative day 20 followed by drug withdrawal as described in *Materials and Methods*. LEW rat recipients that received MHC-matched LEW limbs without TAC served as control. Peripheral blood, skin and muscle tissue samples were collected at 0, 3, 5, 7, 9, 11, and 20 days and assayed for 27 inflammatory mediators using the rat multiplex Luminex™ assay followed by DyBN analysis and DyNA as described in *Materials and Methods*. Panels **(A, B)** show the DyBNs for skin, muscle and peripheral blood (combined) in VCA + TAC and Syngeneic-Control, respectively. Panel **(C)** shows the network complexity (stringency level 0.7) in skin, muscle, and peripheral blood (combined) in VCA+TAC vs. Syngeneic-Control as indicated.

**Figure 9:**
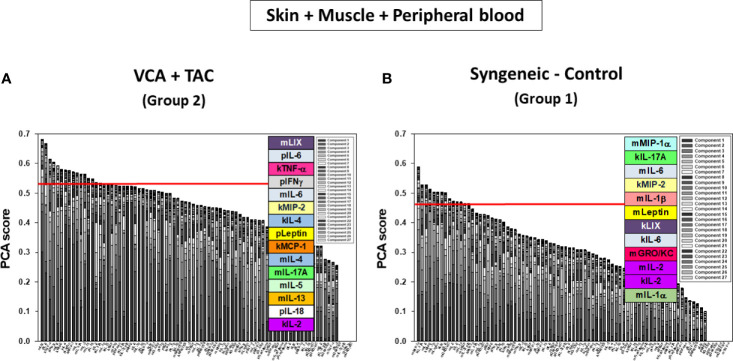
Principal Component Analysis (PCA) suggests differential inflammatory characteristics after VCA + TAC vs. Syngeneic-Control. LEW rat recipients received full MHC-mismatched BN limbs with TAC (1 mg/kg/day, i.p.) until postoperative day 20 followed by drug withdrawal as described in *Materials and Methods*. LEW rat recipients that received MHC-matched LEW limbs without TAC served as control. Peripheral blood, skin and muscle tissue samples were collected at 0, 3, 5, 7, 9, 11, and 20 days and assayed for 27 inflammatory mediators using the rat multiplex Luminex™ assay followed by PCA performed as described in *Materials and Methods.* Figure shows the number of mediators contributing to the top 25% variance of the inflammatory response (above the red line) for skin, muscle, and peripheral blood (combined) in VCA + TAC **(A)** vs. Syngeneic-Control **(B)** as indicated.

DyBN of skin + muscle + peripheral blood (virtual animal) data suggested a complex inflammasome interaction after TAC treatment (Group 2). Peripheral blood Leptin was found as the primary central node with high influence. Peripheral blood LIX was also a central node with fewer connections (**Figure 8A**). However, in syngeneic control animals, fewer inflammatory mediators were involved. Central nodes were peripheral blood Leptin, LIX, and RANTES, as well as skin IL-18 (**Figure 8B**). DyNA of skin + muscle + peripheral blood showed high initial network connectivity/complexity in VCA, resolving partially by POD 7-9 and rising at later time points (**Figure 8C**). However, in the syngeneic control group, network connectivity/complexity was relatively constant over time (**Figure 8C**). Detailed inflammatory networks are depicted in **Supplementary Figure 6**. Finally, PCA suggested a similar overall degree of inflammatory activation in both VCA + TAC and syngeneic transplant, but with different principal characteristics (**Figure 9**).

## Discussion

Our group has previously utilized various machine learning approaches to define the hallmarks and functional modules of acute inflammation in animal models of trauma, sepsis, and transplantation (25, 28, 38, 44–46). In the current study, we examined the singular characteristics of inflammation in the setting of VCA, in order to discern the key drivers of local tissue and systemic inflammation as well as the effect of TAC on such responses. Findings from the present study largely concur with our earlier published studies of protein-level mediators of inflammation in skin and muscle of small animal limb VCA (28) and, as discussed below, with a multitude of studies on various aspects of transplantation. Thus, inflammatory patterns associated with AR are specific for the individual tissue and may serve as superior surrogates for early detection and targeted treatment of AR. Furthermore, we utilized dynamic network inference to unify data across tissues and the circulation, demonstrating the capability of these machine learning tools to unify data across space and time.

### Inferring the Actions of TAC in VCA From Computational Analyses

We used TAC in our study as it is a key drug in the majority of VCA and solid-organ transplant regimens. Studies have shown that TAC suppresses TNF-α-induced MCP-1 and IP-10 expression *via* the inhibition of p38 MAP kinase activation. However, TAC does not suppress TNF-α-induced IL-6 expression (47). In the present studies, we found that inflammation evolves differently in animals treated with TAC vs. syngeneic controls, as reflected and quantified in their network complexity. In syngeneic control animals (Group 1), the inflammatory response is least complex in the peripheral blood, followed by skin and muscle. In contrast, in TAC-treated rats, the exact opposite network complexity pattern was observed. Furthermore, the connectivity/complexity of the systemic inflammatory response (i.e. in the peripheral blood) was much higher in TAC-treated rats; in contrast, dynamic network connectivity/complexity in TAC-treated rats was lower in the muscle of TAC treated rats, and initially higher and later lower in the skin. Taken together, we interpret these results to suggest that TAC has both local and systemic effects, namely that it suppresses inflammation effectively in the muscle but at the cost of increasing systemic inflammation. We hypothesize that the skin, known to be highly immunogenic in the setting of VCA, plays a modulating role, and that TAC impacts that process. These differences in network complexity may be related to the numbers of T cells in each tissue, which are the main cells targeted by TAC to control inflammation in transplant, and a key source of the inflammatory mediators assessed in the present study. This hypothesis is supported by the differential degree of inflammatory infiltrates observed in our histopathologic analyses; future studies will aim to define the cell sub-populations in each tissue.

### Insights Into the Compartmentalized Immune/Inflammatory Response to VCA From Combined *In Vivo* and *In Silico* Studies: Implications for Transplant Rejection

The main achievement of this study was the *in silico* unification of data across skin, muscle, and the peripheral circulation. Combining skin and muscle data yielded an “*in silico* VCA,” while the addition of peripheral blood data arguably yielded a virtual animal (though the typical usage of the term “virtual organism” centers on the use of mechanistic computational modeling, a framework wherein it is possible to simulate behaviors outside of the data used to calibrate the model) (48, 49). We have recently published a detailed, cross-tissue examination of acute inflammation induced by Gram-negative bacterial lipopolysaccharide (LPS) in both wild type mice and mice lacking the main receptor for LPS (Toll-like receptor 4) (40). While that study focused on multiple internal organs as well as the systemic circulation, neither skin nor muscle were assessed, nor were the data integrated to define true cross-tissue inflammatory circuits. In addition, LPS challenge is a simpler experimental paradigm as compared to VCA. The results of the computational tissue integration analyses suggest extensive cross-tissue interactions between skin and muscle, with a key regulatory role for circulating inflammatory mediators such as leptin.

A literature review indicates that currently there are no *in vitro* or *in silico* assays which would comprehensively represent rejection following VCA. The rodent hind limb transplant VCA used in this study is a robust, reliable, and reproducible model that has been validated independently for over 3 decades (13, 50). The rat hind limb is comprised of multiple tissues (skin, muscle, vessel, lymphatics, lymph nodes, bone, cartilage, bone marrow, nerve and adipose tissue) and mimics a clinical VCA such as hand or face transplant. Given its composite tissue nature, this experimental model allows investigation of complex variables in local/systemic inflammation such as surgical trauma, IRI, and the immunogenic effects of multiple allogeneic tissues. Indeed, the stringency of this experimental model was required in order to study the effects of TAC comprehensively. The inflammatory manifestations of surgical trauma or immune rejection occur at the molecular, cellular, tissue and systemic levels. The initial innate immune response involves the release of cytokines, chemokines, and damage-associated molecular proteins (DAMPs) (51), which we have implicated previously as being central to AR using mathematical modeling (17). In the current study, syngeneic transplants (Group 1) were used to study the surgical trauma triggered inflammatory response in the absence of an alloresponse, and allogeneic transplants with TAC (Group 2) helped elucidate the response of surgical trauma induced inflammation and antigen-dependent adaptive immune responses to TAC immunosuppressive therapy. Rejection responses in these experimental groups were compared histopathologically to those of allogeneic transplants without TAC (Group 3, used to study the antigen-dependent immune response that lead to rejection following VCA). Our results suggest that machine learning approaches can elucidate dynamic networks of local and systemic inflammation associated with alloresponses and TAC immunosuppression, which ultimately likely impact AR and possibly also CR.

Unlike in solid organs, antigen presenting cells (APCs) such as Langerhans cell (LC) and keratinocyte components in the skin make this tissue a highly immunogenic barrier to management with conventional immunosuppression. Graft LCs and donor dendritic cells migrate to the local draining lymph nodes (DLNs) after transplantation, where they prime T cells for direct presentation of allogeneic major histocompatibility (MHC) antigens. Later, graft-infiltrating recipient DCs pick up donor major and minor histocompatibility antigens, move back to DLNs, and cross-prime T cells *via* the indirect pathway. The effector T cell response to such LC and DC priming is key to initiation and perpetuation of the host-versus-graft immune response. Early AR in VCA is mediated predominantly by a cross-communication between CD8^+^ T effector and CD4^+^ T helper cells. CD4^+^ T cells also trigger B cell-mediated alloantibody production responsible for late AR, and humoral rejection involving antibody-dependent cellular cytotoxicity or chronic allograft vasculopathy, leading to ischemic graft loss (5, 52). On the other hand, native CD4+ T helper cells can differentiate into Th1 and Th2 subsets (53). Secreted mediators from APCs, such as IL-12, trigger secretion of Th1 and Th2 cytokines such as IFN-*γ*, IL-2, TNFα; IL-4, IL-10, and IL-13 (54), which modulate cellular immunity and also affect macrophage and T cell proliferation and function. Results of PCA in the skin of Group support these mechanisms, with top mediators including IL-12p70, IL-13, and IL-4.

One key mediator inferred by PCA as a primary characteristic of inflammation in Group 2 muscle was IL-17A. Transplant rejection is associated with a predominance of Th1 and Th17 responses over Th2 responses (55–58). In a cohort study of face transplants, it was found that IFN-*γ*, IL-17A, and Th1 as well as Th17 cells were elevated significantly in rejecting grafts (59). Interestingly, a Th1/Th17 pattern was also seen in inflammatory skin conditions such as psoriasis or lupus, possibly because Leptin suppresses regulatory T cells and enhances Th17 cells (60). IL-4-secreting Th cells were found to be protective in kidney transplant recipients (61). In addition, tissue trauma causes suppression of Th1 responses and induces Th2 responses with selective cellular suppression (62).

Using DyBN inference in our experimental VCA setting, we identified IL-1α, IL-18, IL-1β, and IL-4 as principal characteristics associated with, or potential drivers of, transplant rejection. Interestingly, IL-1α is an allograft-induced DAMP (63) that has been implicated in IL-17A production in an experimental model of transplant rejection (64). Interleukins-1β and -18 implicate the NLRP3 inflammasome, known to be involved in transplant rejection (65). Another cytokine associated with transplant rejection is IL-4, possibly due to its role in promoting M2 macrophage differentiation and function (66). We also found evidence suggesting that Leptin and IL-18 were leading central nodes and potential drivers interacting with pyroptosis and NLRP inflammasome in the skin, whereas Leptin was also involved in a pro-healing/M2 program *via* IL-4, IL-5, and IL-13 in muscle. Leptin is secreted by adipocytes, the placenta, and the stomach (67). The cytokine receptor family includes Leptin receptors, (68). Leptin is known to increase acutely as a response to inflammatory triggers (69). Leptin levels, as well as IL-1β and IL-6, increase in burn injury, which suggests that Leptin may be related to pro-inflammatory cytokines (including a Th1 phenotype shift) (70). Indeed, Leptin injection reverses the immunosuppressive effect of burn injury (71). Leptin levels also increase after sepsis and septic shock, probably as a host mechanism to defend against bacterial infection (72). Leptin modulates nonspecific cytokine responses mediated by macrophages and is required for complement mediated activity of neutrophils (73). Leptin promotes VEGF, IL-6. and PGE2 production (74). Most importantly, and relevant to our current study, high concentrations of Leptin in serum constitute an independent risk factor for the development of AR in kidney transplants (75).

DyNA suggests that inflammatory network complexity was decreased in POD 5-9 in skin and muscle; however, peripheral blood had intermittent inflammation from POD Days 0-20. Based on DyBN inference, the surgical inflammation following syngeneic transplants (Group 1) showed a similar systemic response as with allogeneic transplants receiving TAC, but the inflammatory response was simpler. DyNA suggested that inflammatory networks in the skin and muscle were more complex at earlier time points, whereas the network in peripheral blood was more complex in the middle time points. In the VCA + TAC setting (Group 1), we found IL-1α in skin, IL-18 in muscle, and Leptin in peripheral blood as central nodes in DyBN (data not shown). DyNA showed decreasing complexity in peripheral blood, contrary to complexity in skin and muscle were increasing over time. The impact of IL12p70 that has already been shown to be a potential early diagnostic marker for AR was not as pronounced in the TAC-treated animals as compared to the syngeneic or allogeneic transplants without TAC. Interferon-γ and IL-4 had a high ranking in all groups, indicating a non-specific effect in inflammation. In contrast, IL-1α and IL-18 exhibited an expression profile which indicates a possible key role in VCA rejection and makes these cytokines interesting candidates for therapeutic interventions (28).

Our results in individual tissues are in agreement with prior studies from our group and others utilizing various statistical approaches to define predictors of AR in the context of experimental VCA. We have used multiplexed analysis technology to study protein levels of 27 inflammatory mediators in skin and muscle biopsies as well as peripheral blood samples from syngeneic and allogeneic limb transplants without immunosuppression and limb allografts treated with TAC, with the goal of creating predictive statistical models. We examined the levels of these inflammatory analytes at different postoperative days, with a focus on the early postoperative phase where no histological alterations were observed in any of the three groups (POD 3 and 5) (28). Initially, non-parametric univariate analysis of the inflammatory mediators from skin and muscle were performed. Five inflammatory mediators (GM-CSF, IL1-α, IL-4, IL-12p70, IL-5, TNF-α) were significantly different at least in one group (adjusted p<0.05; Kruskal-Wallis test [KW]) in both skin and muscle. IL-12p70 and TNF-α were highly significantly different in the allograft versus the tacrolimus-treated animals (28). For identification of inflammatory mediators with the highest predicting value, at early time points, multivariate analyses were performed, suggesting that GM-CSF, IL-4, IL-12p70, IL-5, and TNF-α could be promising biomarkers of AR. The prediction accuracy of these models within skin was 87.1% and in muscle 100%, and a pairwise multivariate logistic regression analysis between the study groups (Group 1 vs. Group 2, Group 2 vs. Group 1 and Group 1 vs. Group 1) and applying a leave-one-out cross validation strategy resulted in an area under curve (AUC) from receiver operating characteristics (ROC) for skin of 0.5, 0.69, and 0.86 and for muscle of 1.0, 1.0, and 1.0) (28). In related work, a recent study in syngeneic and allogeneic groin flap VCAs analyzed gene and protein mediators on POD 2 and 5 using quantitative real-time PCR and multiplex analyses to delineate whether injury-induced inflammation triggers allograft rejection. IL-18, IFN*γ*, IP-10/CXCL10, RANTES/CCL5, Gro-α/CXCL1, and IL-10 were upregulated in the allogeneic group while remaining unchanged in the syngeneic group (76).

### Limitations of This Study

A primary study limitation was the short duration of follow-up of animals for 20 days for multiplex collection/analyses of tissue and peripheral blood samples. However, our goal was to identify the nature, pattern, and timeline of inflammatory mediator expression and complex network interactions occurring *early* after VCA during the prime time window that encompasses inflammatory responses secondary to surgical trauma, ischemia/reperfusion injury and AR. Other important limitations are those inherent to the computational methods employed. For example, DyBN inference assumes a fixed network structure over the full time course; while this allows for the inference of central nodes that feedback upon themselves and affect downstream nodes, the assumption of a fixed network structure may be incorrect or insufficient. In contrast, while DyNA allows for network inference over defined time intervals, this method does not allow for the direct inference of nodes involved in self-feedback. Hence, these methods were utilized in concert.

## Conclusions

In conclusion, this study extends initial work characterizing dynamic inflammatory changes in rats undergoing VCA (27, 28). We have defined protein-level dynamic networks of inflammation in skin, muscle, and the systemic circulation in the context of experimental VCA and gained insights into the potential impact of TAC. Together, we believe that our comprehensive, computational, and systems biology analysis of the production and dynamic interactions of key mediators during the onset, evolution, and progression of inflammation/rejection after VCA with and without the influence of TAC could define novel strategies for non-invasive surrogate monitoring, graft specific immunomodulation, and optimization of immunologic and functional graft survival outcomes.

## Data Availability Statement

The datasets presented in this study can be found in online repositories. The names of the repository/repositories and accession number(s) can be found in the article/**Supplementary Material**.

## Ethics Statement

The animal study was reviewed and approved by Institutional Animal Care and Use Committee (IACUC) of the University of Pittsburgh and the Department of Defense Animal Care and Utilization Review Office (ACURO).

## Author Contributions

AA performed animal experiments and wrote manuscript. RZ performed computational analyses and wrote manuscript. DB performed Luminex analyses. JY performed Luminex analysis. FE-D performed Luminex analysis. VE performed animal experiments. LD performed animal experiments. ZZ performed animal experiments. HS performed animal experiments. VG conceived studies and wrote manuscript. YV conceived studies and wrote manuscript. All authors contributed to the article and approved the submitted version.

## Funding

This work was supported by Dept. of Defense grant W81 XWH-15-1-0336 (YV, PI).

## Conflict of Interest

The authors declare that the research was conducted in the absence of any commercial or financial relationships that could be construed as a potential conflict of interest.
