# Supplementary material for: The Effects of Tacrolimus on Tissue-Specific, Protein-Level Inflammatory Networks in Vascularized Composite Allotransplantation
Source: Front Immunol. 2021 May 4;12:591154. doi: 10.3389/fimmu.2021.591154 (PMC8129572; doi:10.3389/fimmu.2021.591154)
Supplement: Supplementary file 1 [file DataSheet_1.pdf]

Supplementary Material

Treatment	Animal ID	time-points	Animal ID	time-points	Animal ID	time-points
**syng Plasma**	Rat 1-4	**d0, d5, d9, d20**	Rat 5-8	**d3, d7, d 11**		
**syng Skin**	Rat 1-4	**d0, d5, d9, d20**	Rat 5-8	**d3, d7, d 11**		
**syng Muscle**	Rat 1-4	**d0, d5, d9, d20**	Rat 5-8	**d3, d7, d 11**		
**total**	**n=4**	**4 tps**	**n=4**	**3 tps**		
**VCA+ TAC Plasma**	Rat 9-12	**d0, d5, d9**	Rat 13-16	**d3, d7, d 11**	Rat 17-20	**d20**
**VCA+ TAC Skin**	Rat 9-12	**d0, d5, d9**	Rat 13-16	**d3, d7, d 11**	Rat 17-20	**d20**
**VCA+ TAC Muscle**	Rat 9-12	**d0, d5, d9**	Rat 13-16	**d3, d7, d 11**	Rat 17-20	**d20**
**total**	**n=4**	**3 tps**	**n=4**	**3 tps**	**n=4**	**1 tp**

Supplementary Figures and Tables

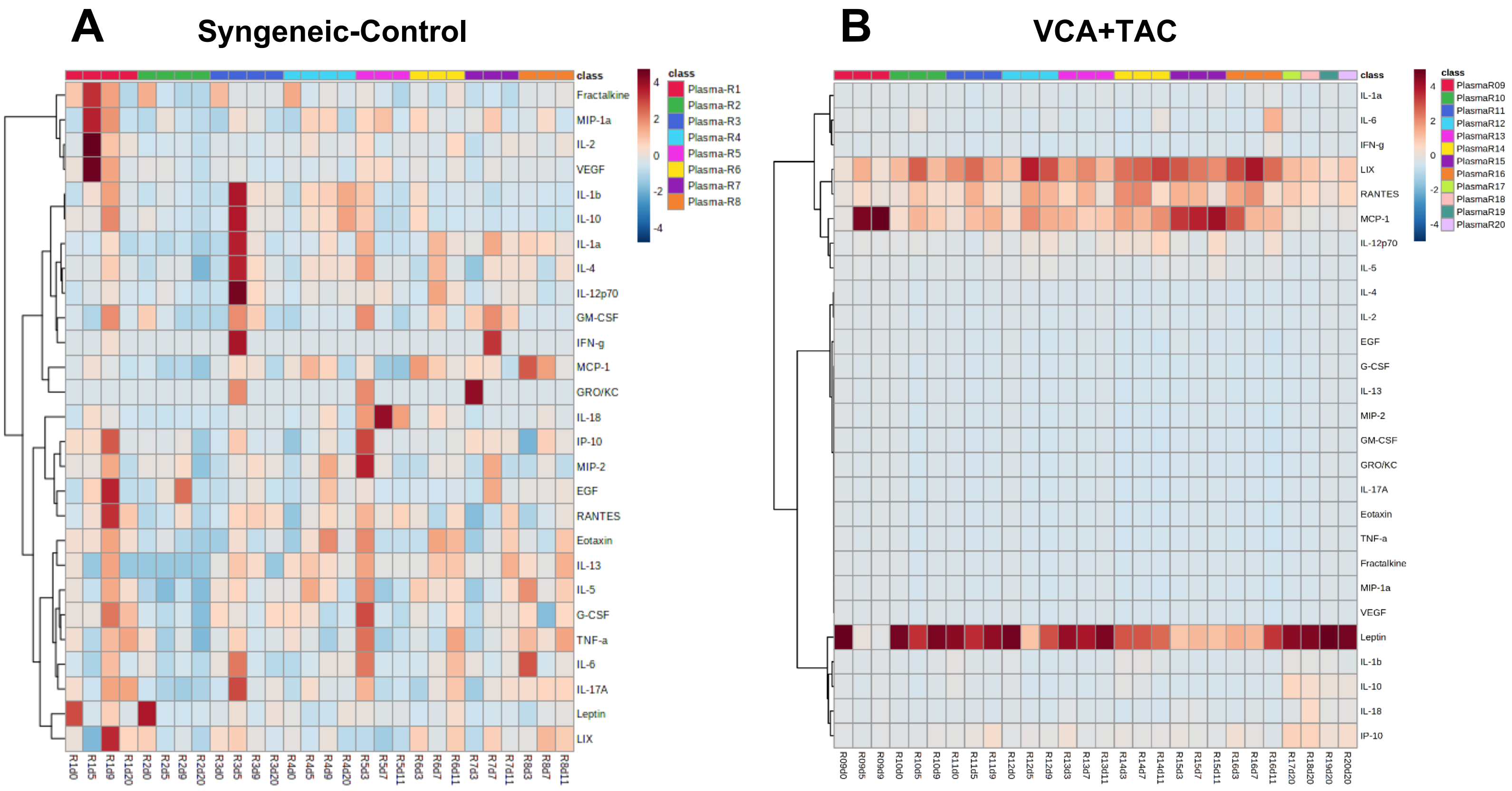

Supplementary Figure 1. Hierarchical Clustering Analysis of all plasma data broken down by rats. (A) Syngeneic-Control. **(B)** VCA+TAC.

Supplementary Figure 2. Time-dependent release of inflammatory mediators in rats undergoing VCA +TAC vs. Syngeneic Control. LEW rat recipients received full MHC-mismatched BN limbs with TAC (1 mg/kg/day, i.p.) until postoperative day 20 followed by drug withdrawal as described in *Materials and Methods*. LEW rat recipients that received MHC-matched LEW limbs without TAC served as control. Peripheral blood, skin and muscle tissue samples were collected at 0, 3, 5, 7, 9, 11, and 20 days and assayed for 27 inflammatory mediators using the rat multiplex Luminex™ assay as described in *Materials and Methods*. Cytokine concentrations are expressed in pg/mg protein (skin and muscle) or pg/ml (peripheral blood) + SEM as indicated (VCA + TAC vs. Syngeneic-Control, analyzed by Two-Way ANOVA, followed by Holm-Sidak post-hoc test, $P<0.05$).

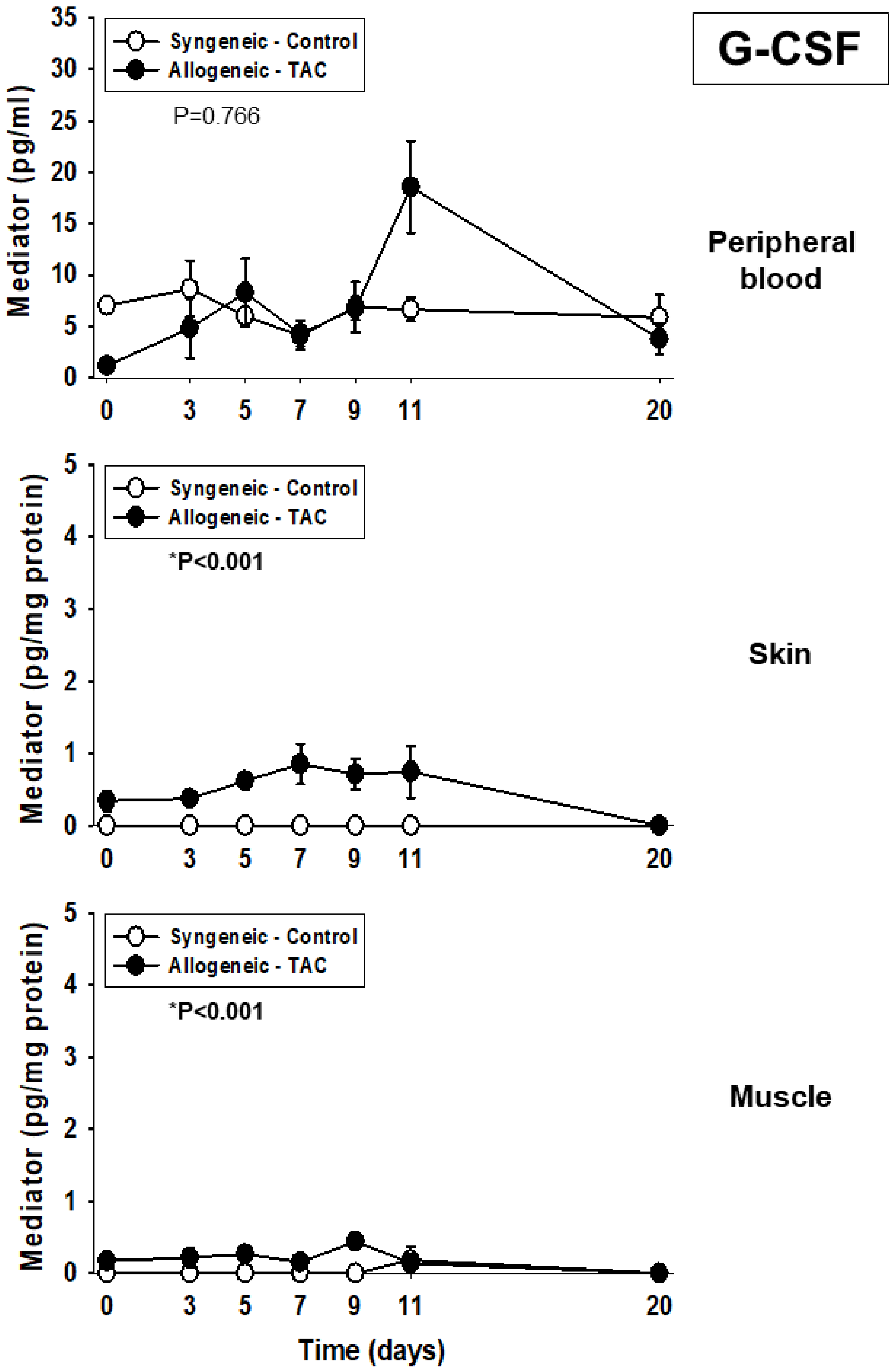
G-CSF
Syngeneic - Control
Allogeneic - TAC
P=0.766
Peripheral blood
Mediator (pg/ml)
35
30
25
20
15
10
5
0
0 3 5 7 9 11 20
Syngeneic - Control
Allogeneic - TAC
*P<0.001
Skin
Mediator (pg/mg protein)
5
4
3
2
1
0
0 3 5 7 9 11 20
Syngeneic - Control
Allogeneic - TAC
*P<0.001
Muscle
Mediator (pg/mg protein)
5
4
3
2
1
0
0 3 5 7 9 11 20
Time (days)

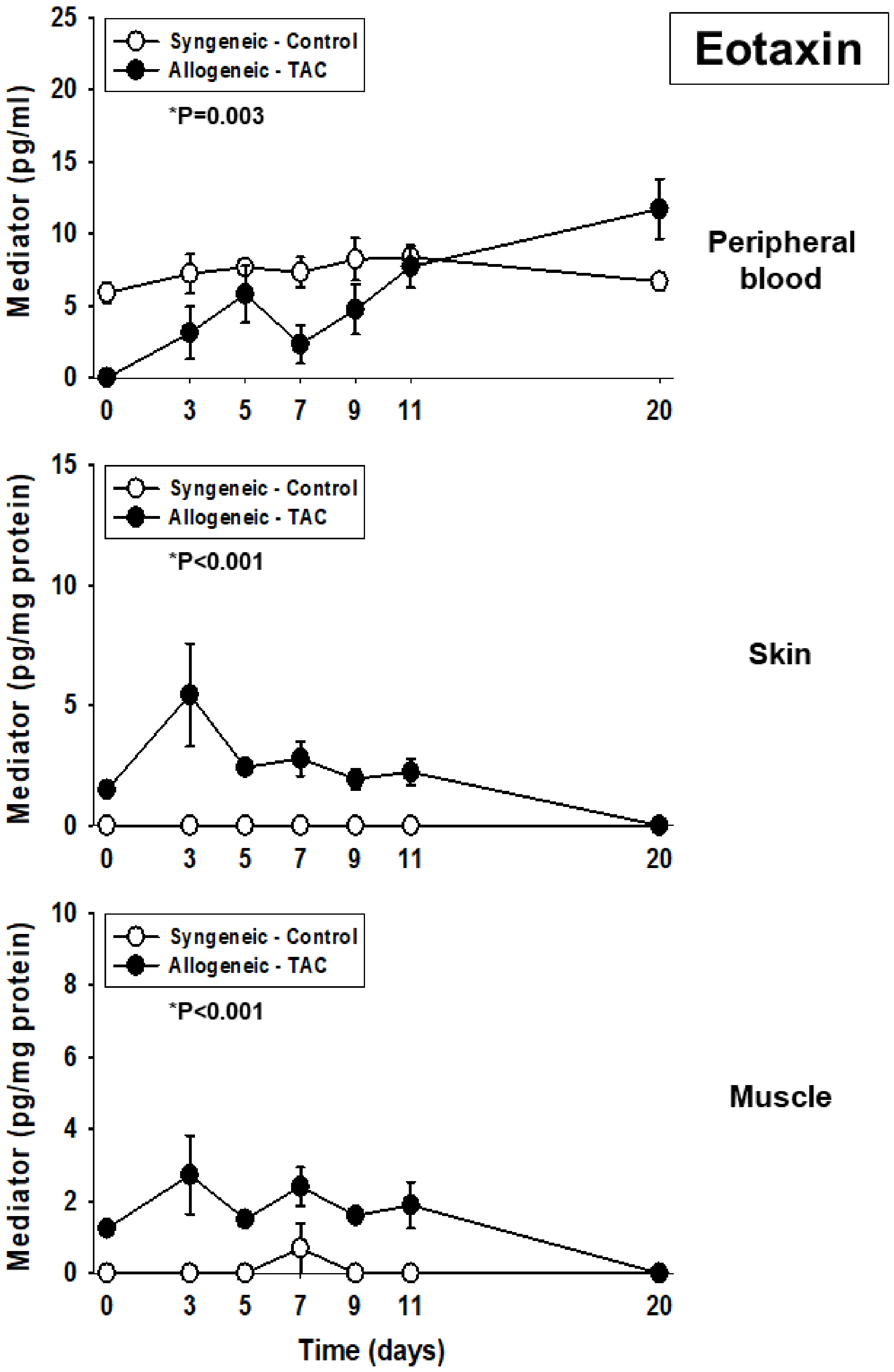
Eotaxin
Syngeneic - Control
Allogeneic - TAC
*P=0.003
Mediator (pg/ml)
25
20
15
10
5
0
0
3
5
7
9
11
20
Peripheral blood
Syngeneic - Control
Allogeneic - TAC
*P<0.001
Mediator (pg/mg protein)
15
10
5
0
Skin
Syngeneic - Control
Allogeneic - TAC
*P<0.001
Mediator (pg/mg protein)
10
8
6
4
2
0
Muscle
Time (days)

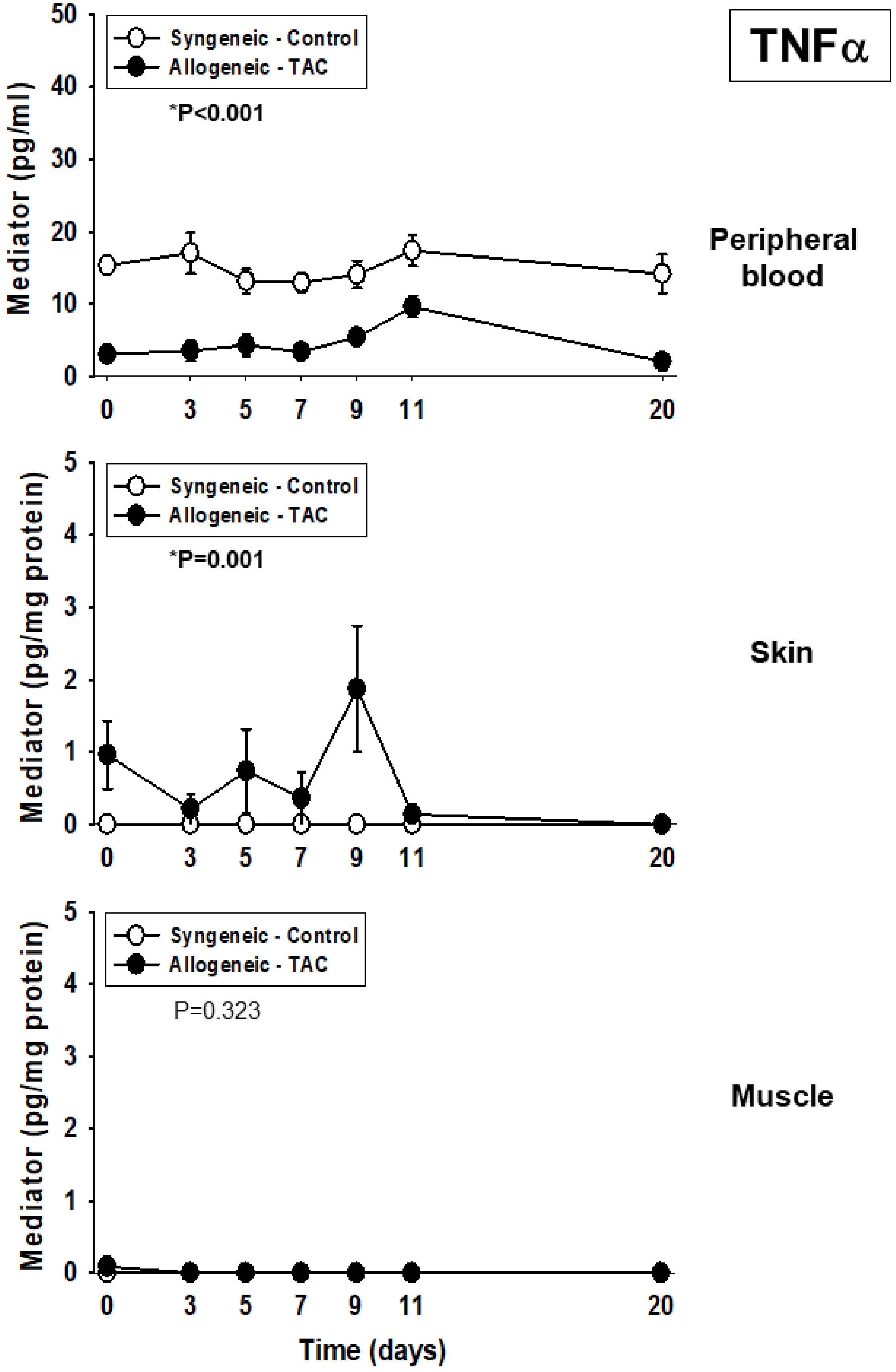
TNFα
Syngeneic - Control
Allogeneic - TAC
*P<0.001
Mediator (pg/ml)
50
40
30
20
10
0
0
3
5
7
9
11
20
Peripheral blood
Syngeneic - Control
Allogeneic - TAC
*P=0.001
Mediator (pg/mg protein)
5
4
3
2
1
0
Skin
Syngeneic - Control
Allogeneic - TAC
P=0.323
Mediator (pg/mg protein)
Muscle
Time (days)

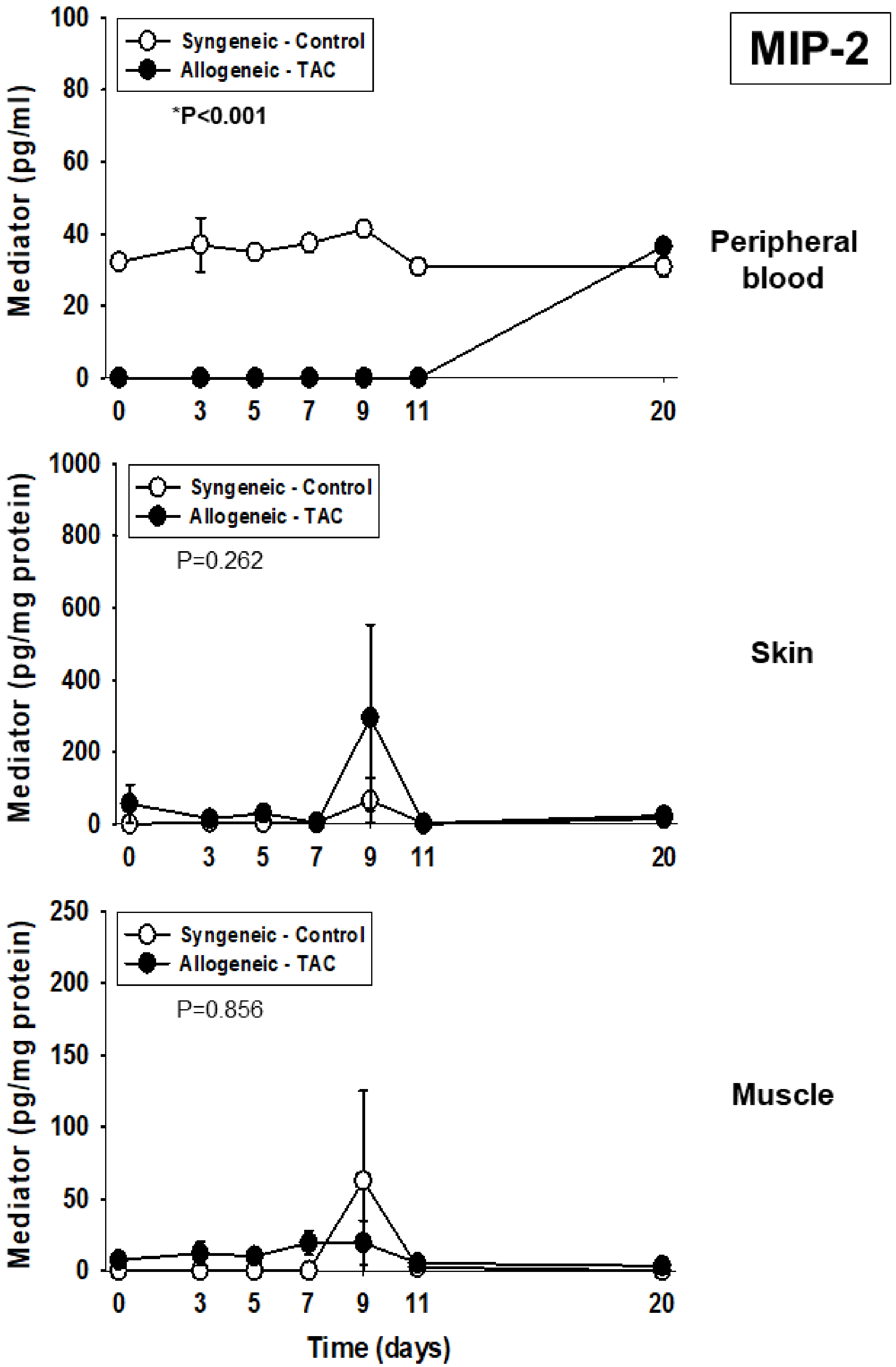
MIP-2
Syngeneic - Control
Allogeneic - TAC
*P<0.001
Mediator (pg/ml)
100
80
60
40
20
0
0 3 5 7 9 11 20
Peripheral blood
Syngeneic - Control
Allogeneic - TAC
P=0.262
Mediator (pg/mg protein)
1000
800
600
400
200
0
0 3 5 7 9 11 20
Skin
Syngeneic - Control
Allogeneic - TAC
P=0.856
Mediator (pg/mg protein)
250
200
150
100
50
0
0 3 5 7 9 11 20
Time (days)
Muscle

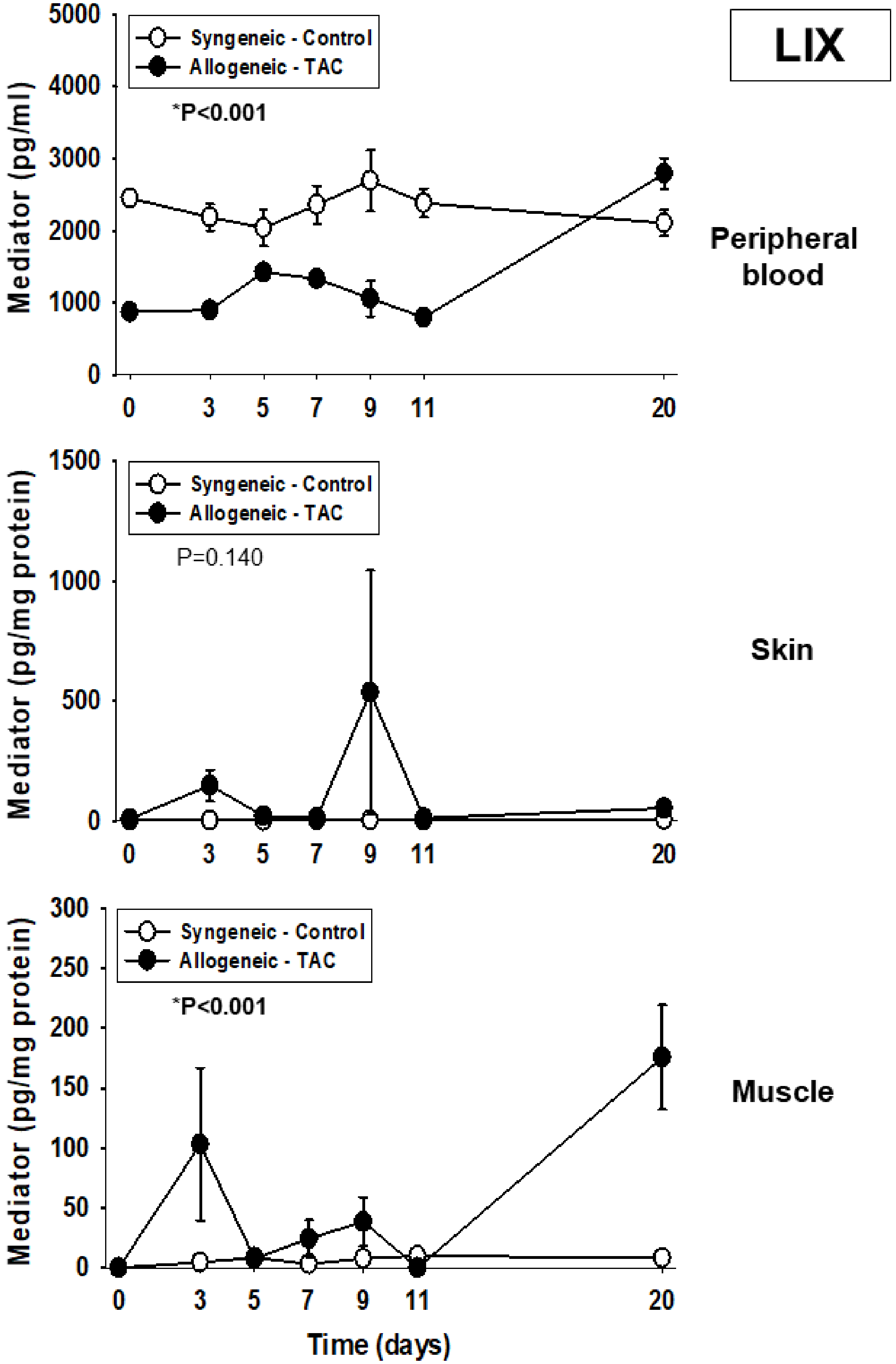

LIX
Syngeneic - Control
Allogeneic - TAC
*P<0.001
Mediator (pg/ml)
5000
4000
3000
2000
1000
0
0
3
5
7
9
11
20
Peripheral blood
Syngeneic - Control
Allogeneic - TAC
P=0.140
Mediator (pg/mg protein)
1500
1000
500
0
0
3
5
7
9
11
20
Skin
Syngeneic - Control
Allogeneic - TAC
*P<0.001
Mediator (pg/mg protein)
300
250
200
150
100
50
0
0
3
5
7
9
11
20
Muscle
Time (days)

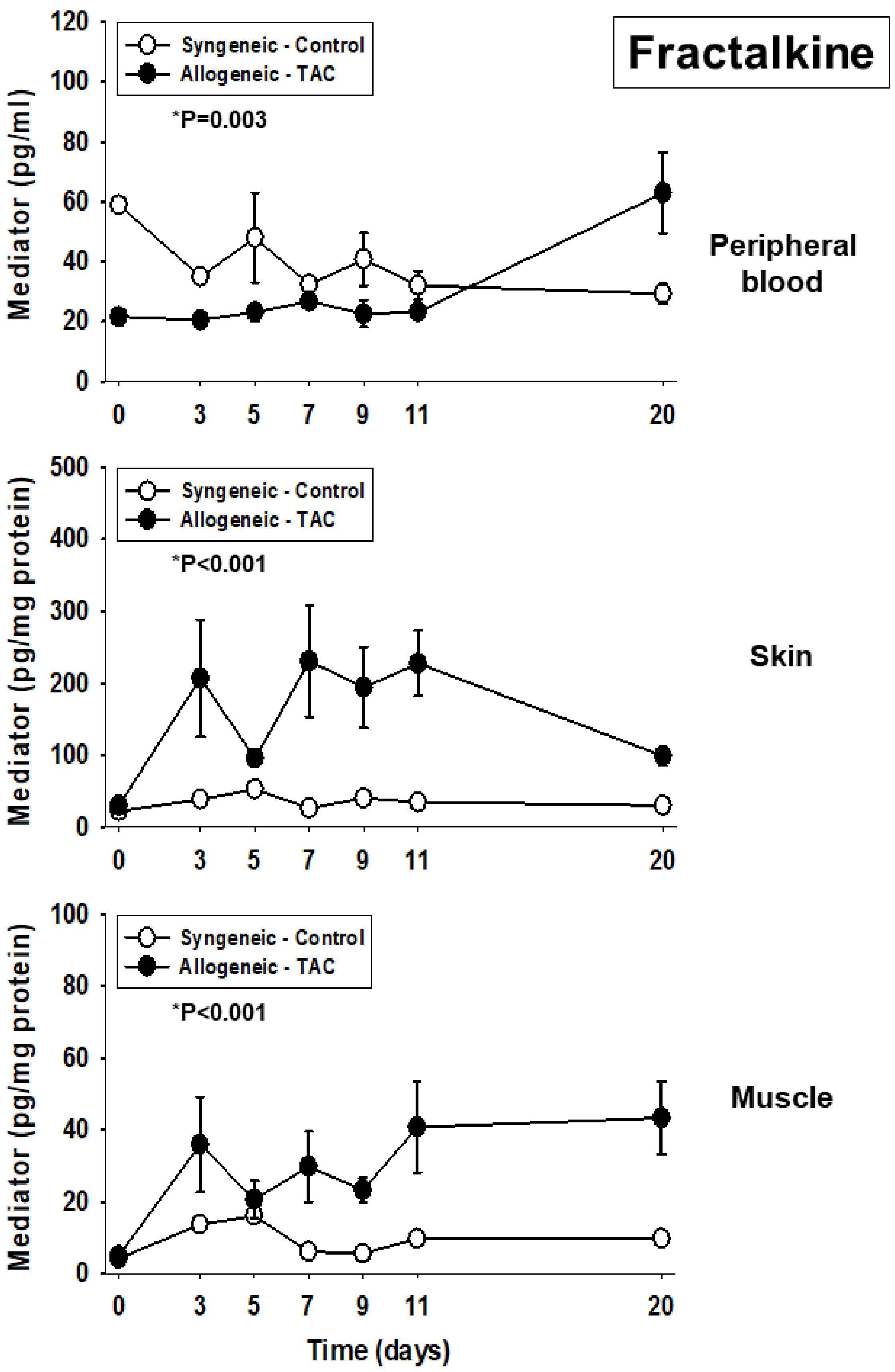

Fractalkine
Syngeneic - Control
Allogeneic - TAC
*P=0.003
Mediator (pg/ml)
120
100
80
60
40
20
0
0
3
5
7
9
11
20
Peripheral blood
Syngeneic - Control
Allogeneic - TAC
*P<0.001
Mediator (pg/mg protein)
500
400
300
200
100
0
Skin
Syngeneic - Control
Allogeneic - TAC
*P<0.001
Mediator (pg/mg protein)
100
80
60
40
20
0
Muscle
Time (days)

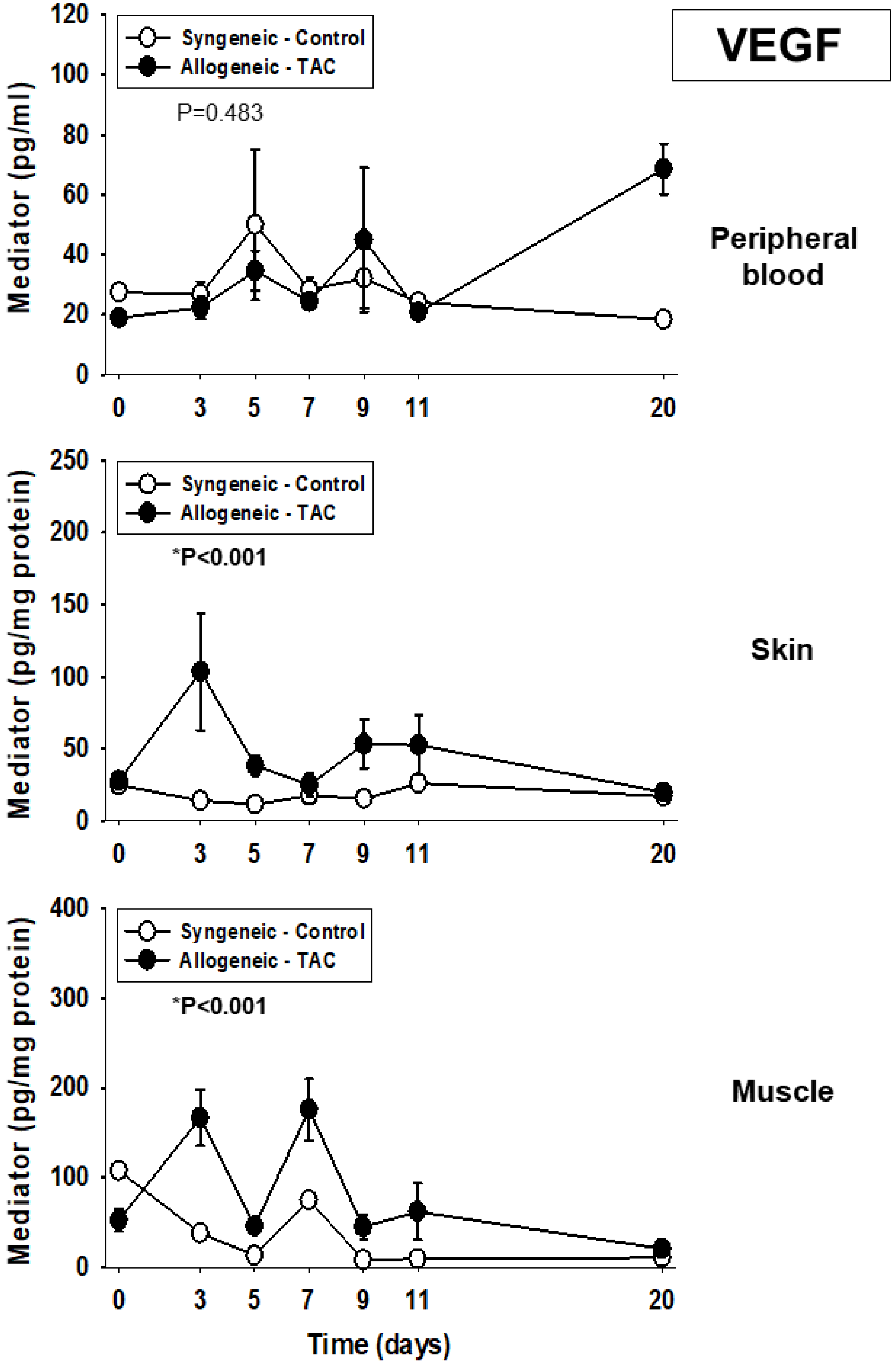
VEGF
Syngeneic - Control
Allogeneic - TAC
P=0.483
Mediator (pg/ml)
Peripheral blood
*P<0.001
Mediator (pg/mg protein)
Skin
Muscle
Time (days)
0
3
5
7
9
11
20

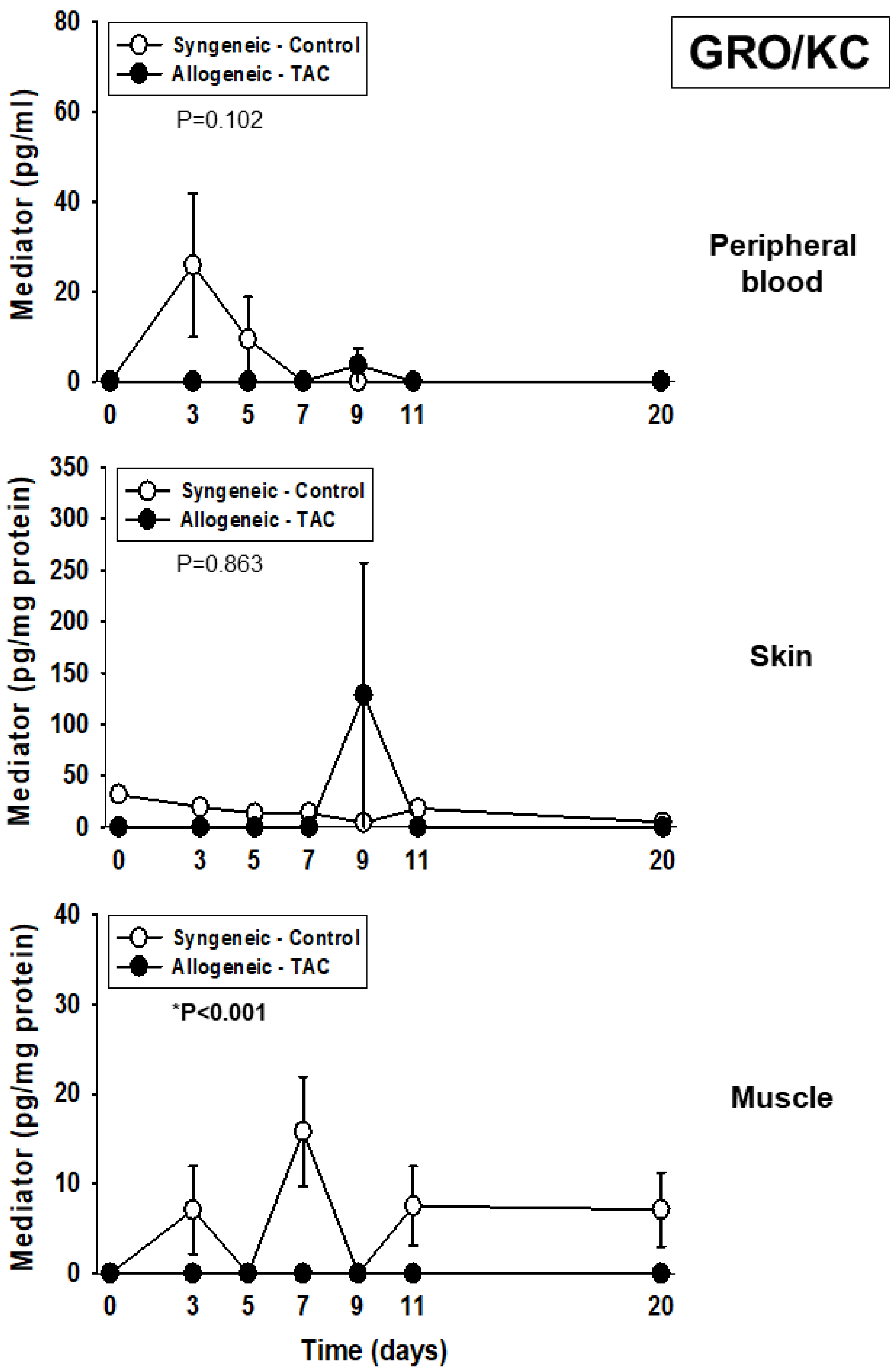
GRO/KC
Syngeneic - Control
Allogeneic - TAC
P=0.102
Mediator (pg/ml)
Peripheral blood
P=0.863
Mediator (pg/mg protein)
Skin
*P<0.001
Muscle
Time (days)

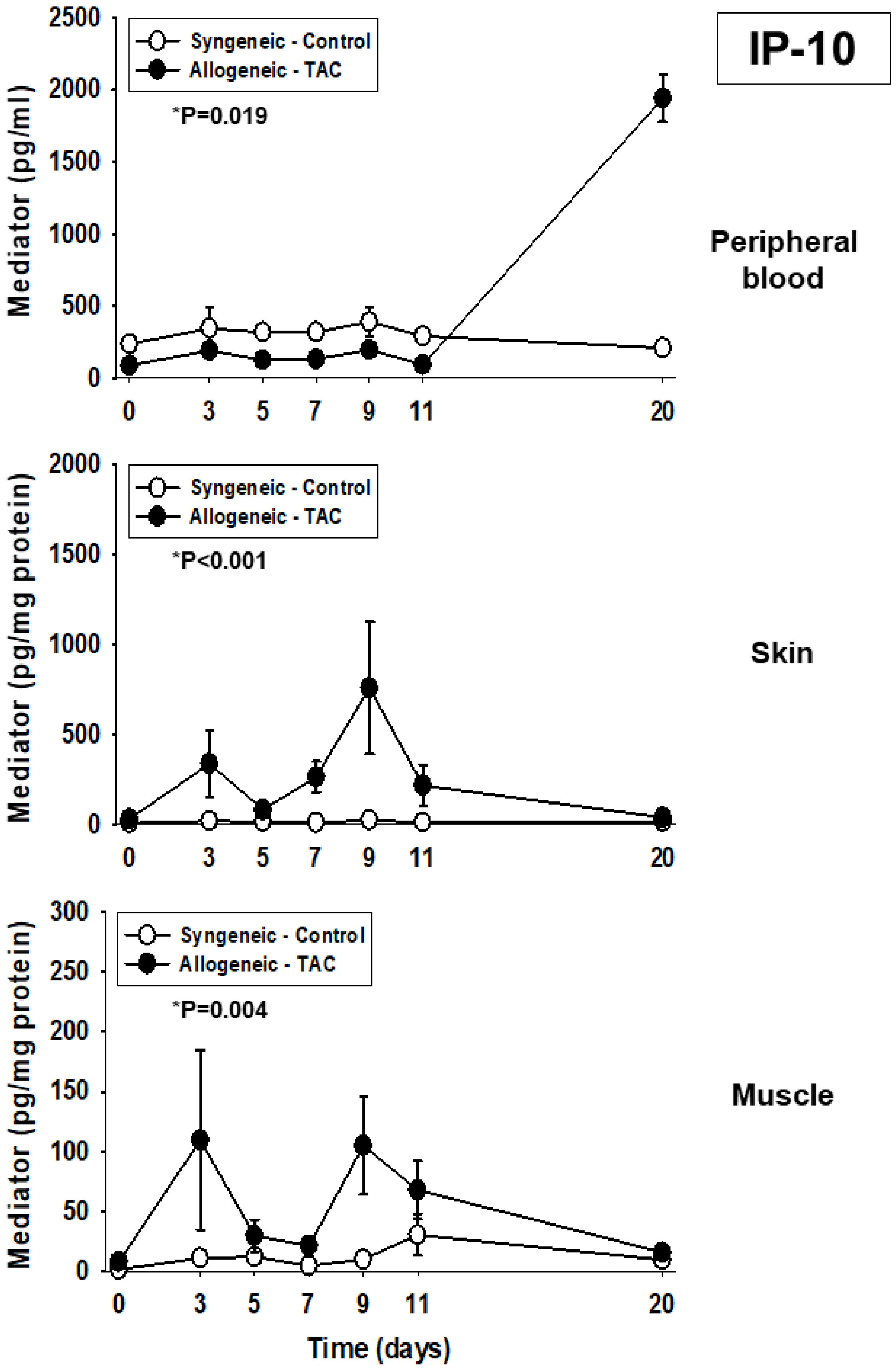
IP-10
Mediator (pg/ml)
2500
2000
1500
1000
500
0
Syngeneic - Control
Allogeneic - TAC
*P=0.019
0
3
5
7
9
11
20
Peripheral blood
Mediator (pg/mg protein)
2000
1500
1000
500
0
Syngeneic - Control
Allogeneic - TAC
*P<0.001
0
3
5
7
9
11
20
Skin
Mediator (pg/mg protein)
300
250
200
150
100
50
0
Syngeneic - Control
Allogeneic - TAC
*P=0.004
0
3
5
7
9
11
20
Time (days)
Muscle

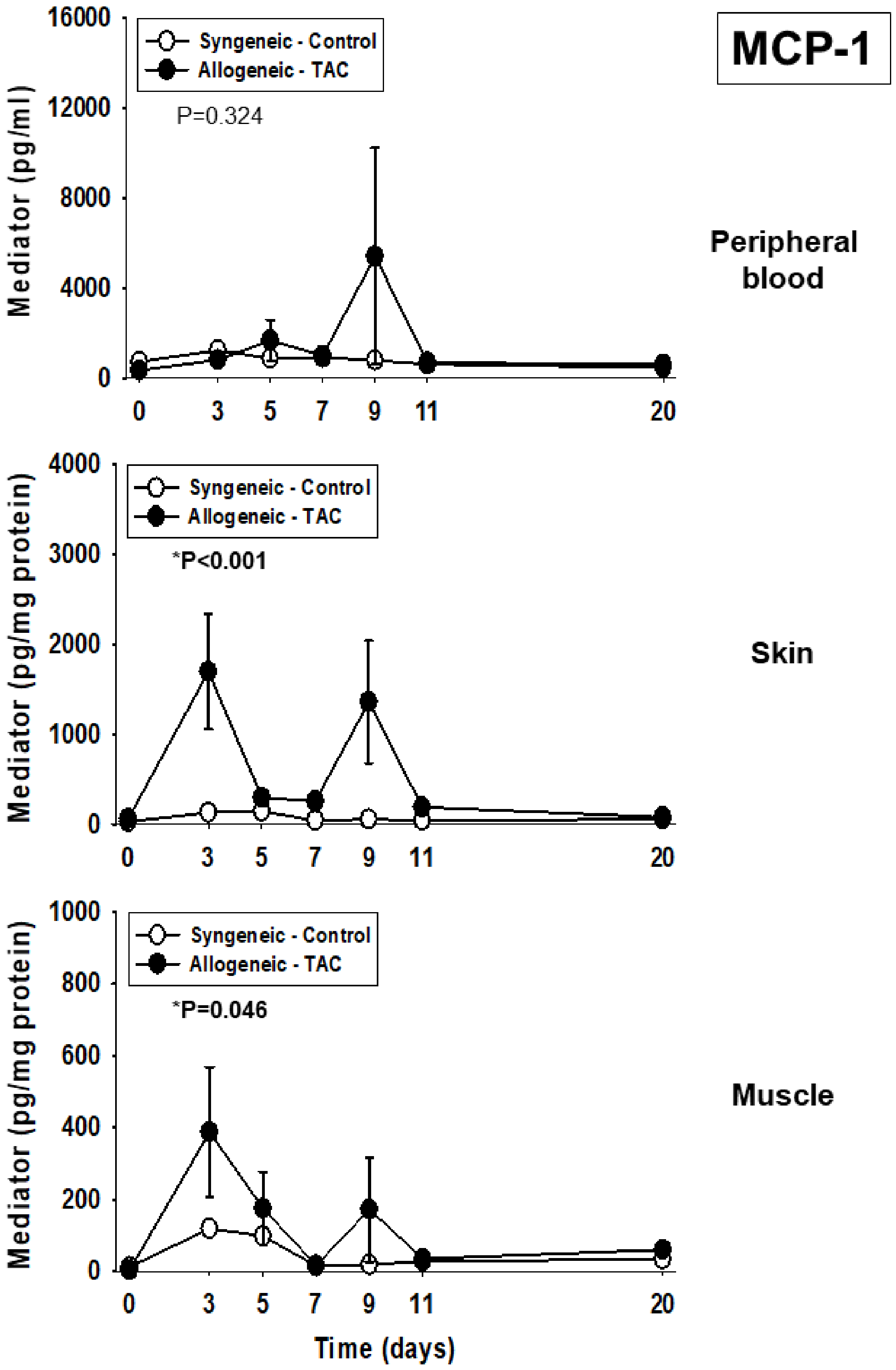
MCP-1
Syngeneic - Control
Allogeneic - TAC
P=0.324
Mediator (pg/ml)
16000
12000
8000
4000
0
0
3
5
7
9
11
20
Peripheral blood
Syngeneic - Control
Allogeneic - TAC
*P<0.001
Mediator (pg/mg protein)
4000
3000
2000
1000
0
Skin
Syngeneic - Control
Allogeneic - TAC
*P=0.046
Mediator (pg/mg protein)
1000
800
600
400
200
0
Muscle
Time (days)

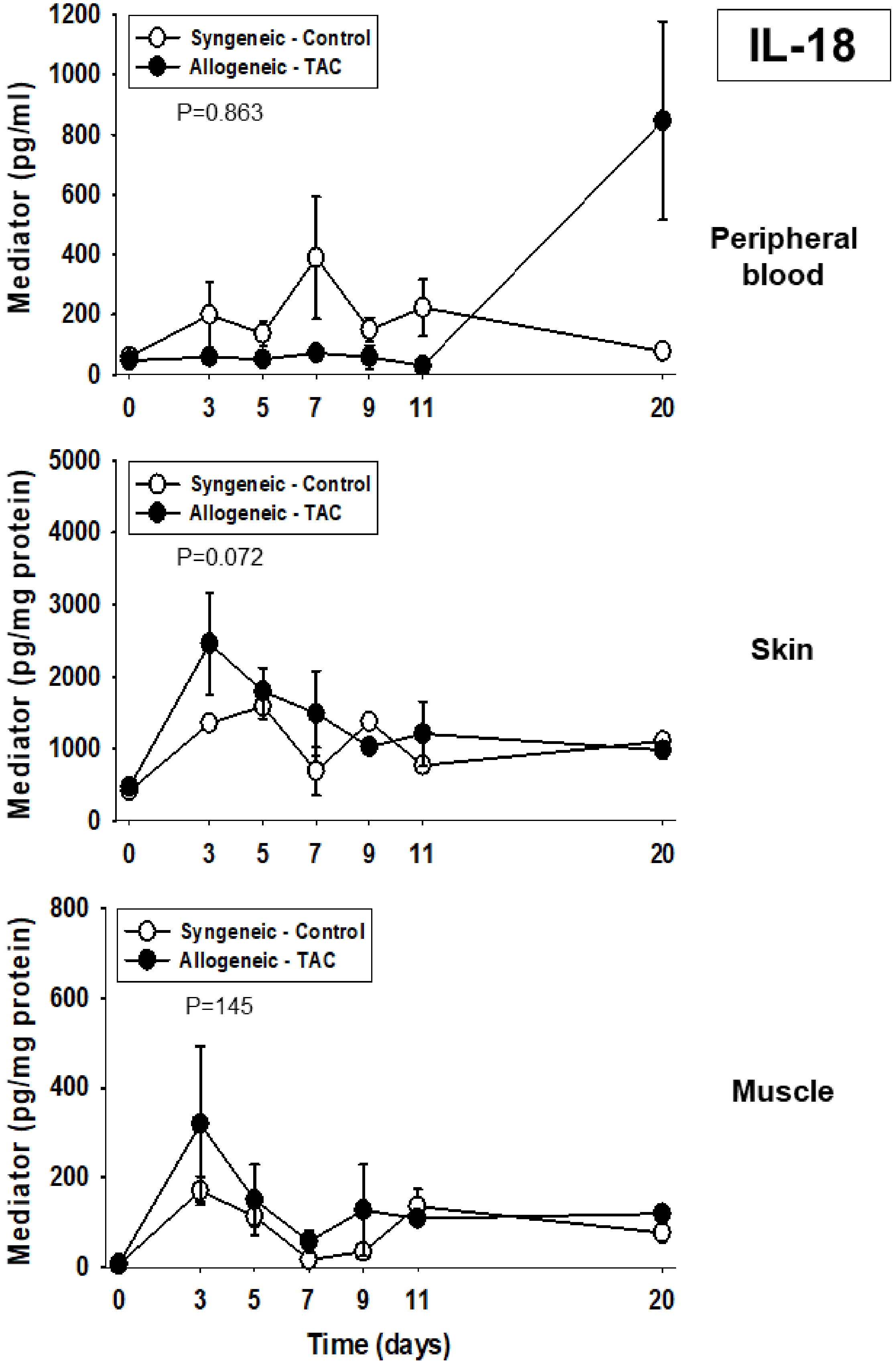
IL-18
Peripheral blood
Skin
Muscle
Syngeneic - Control
Allogeneic - TAC
P=0.863
P=0.072
P=145
Mediator (pg/ml)
Mediator (pg/mg protein)
Mediator (pg/mg protein)
1200
1000
800
600
400
200
0
5000
4000
3000
2000
1000
0
800
600
400
200
0
0
3
5
7
9
11
20
Time (days)

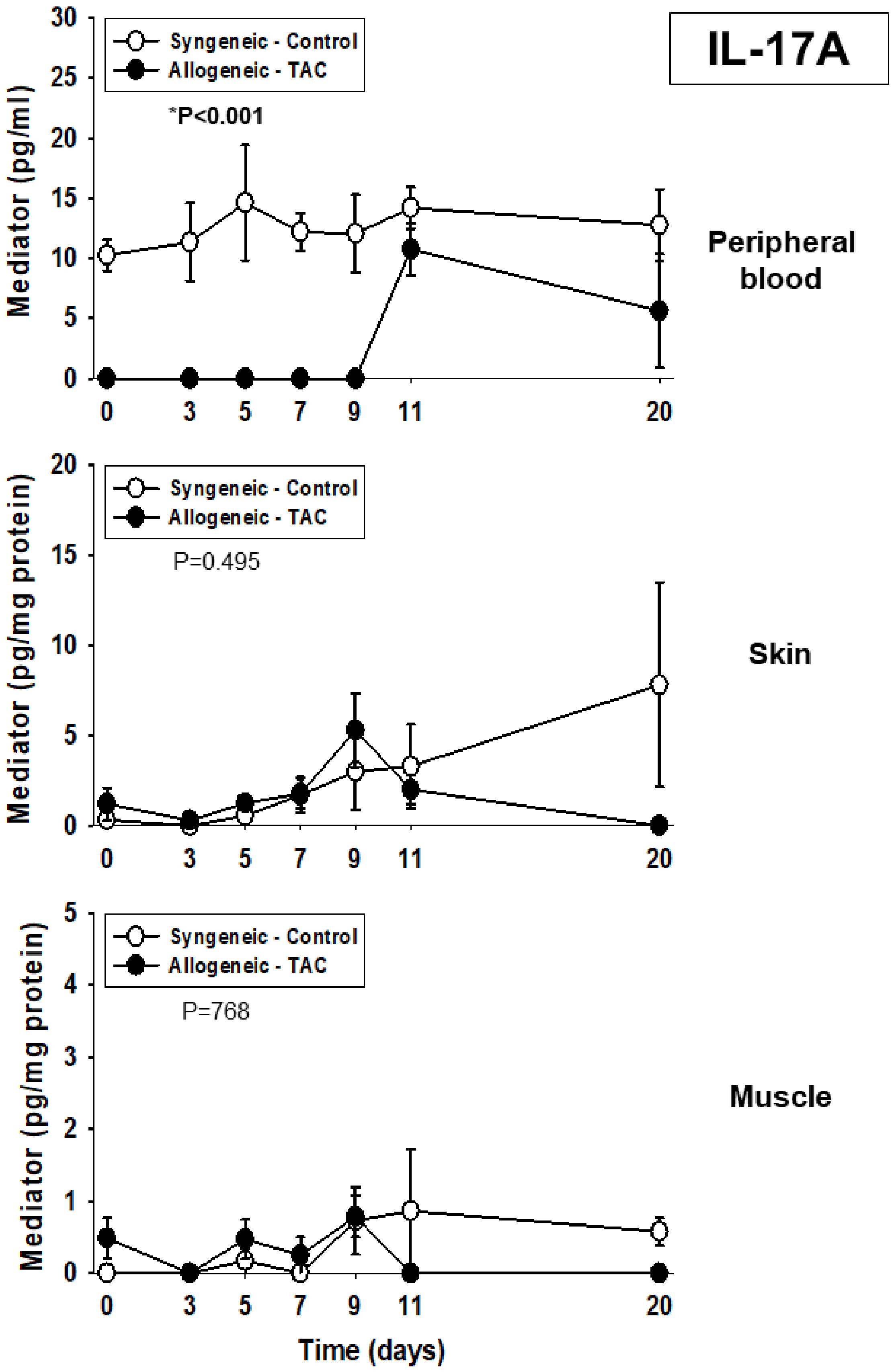
IL-17A
Syngeneic - Control
Allogeneic - TAC
*P<0.001
Mediator (pg/ml)
Peripheral blood
P=0.495
Mediator (pg/mg protein)
Skin
P=768
Muscle
Time (days)

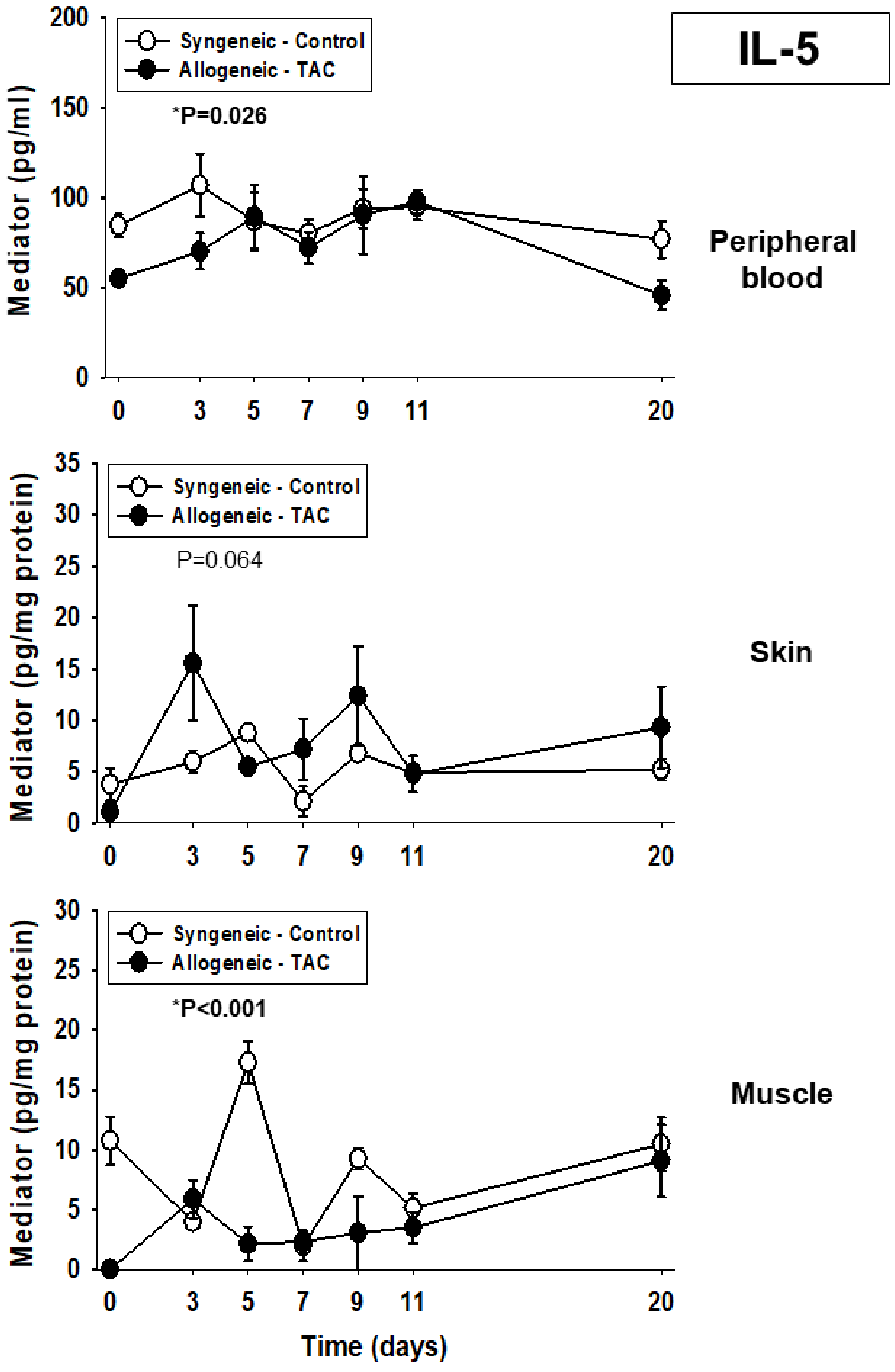
IL-5
Syngeneic - Control
Allogeneic - TAC
*P=0.026
Mediator (pg/ml)
200
150
100
50
0
0
3
5
7
9
11
20
Peripheral blood
Syngeneic - Control
Allogeneic - TAC
P=0.064
Mediator (pg/mg protein)
35
30
25
20
15
10
5
0
Skin
Syngeneic - Control
Allogeneic - TAC
*P<0.001
Mediator (pg/mg protein)
30
25
20
15
10
5
0
Muscle
Time (days)

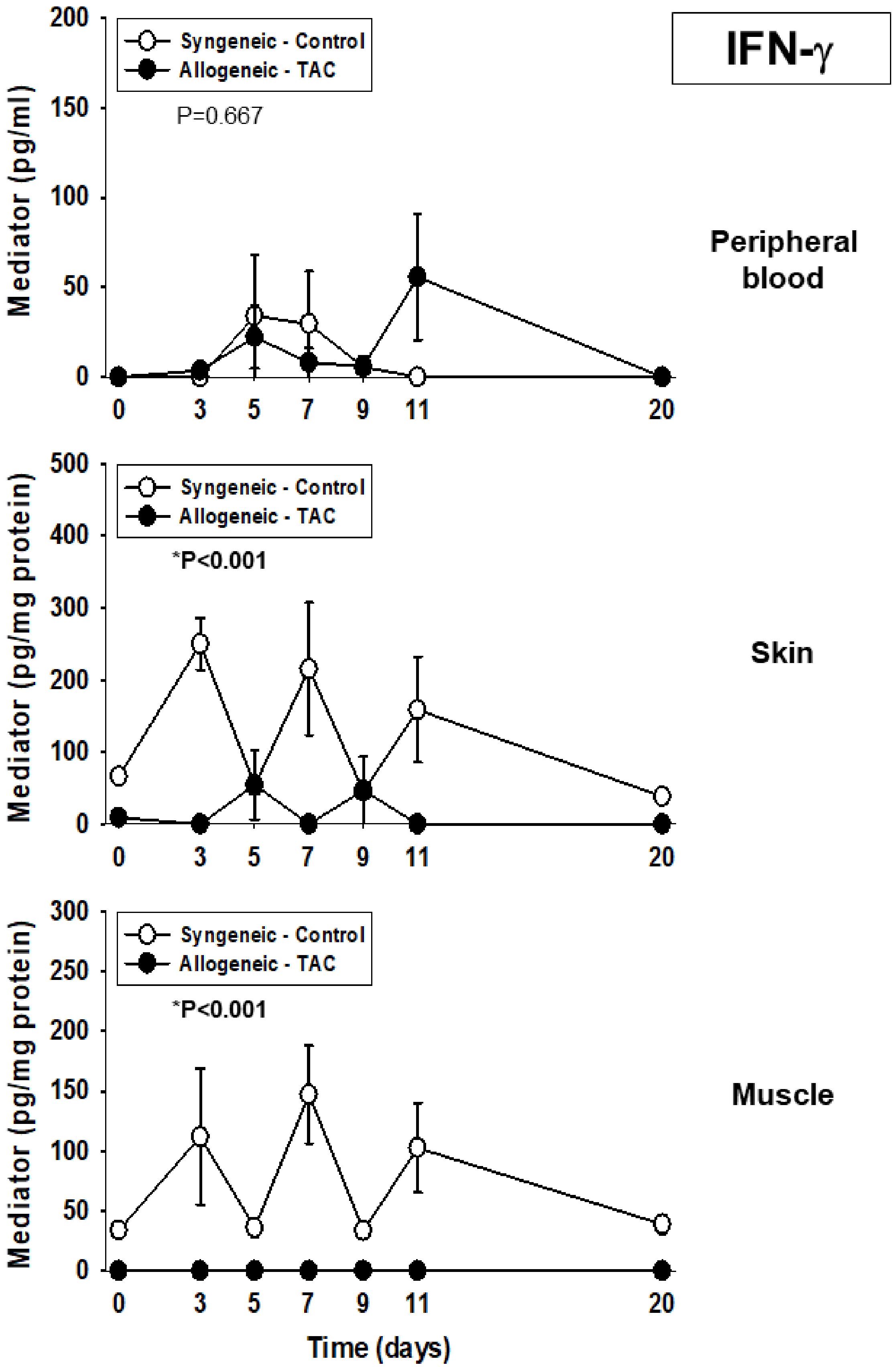
IFN-γ
Syngeneic - Control
Allogeneic - TAC
P=0.667
Peripheral blood
*P<0.001
Skin
*P<0.001
Muscle
Mediator (pg/ml)
Mediator (pg/mg protein)
0
3
5
7
9
11
20
Time (days)

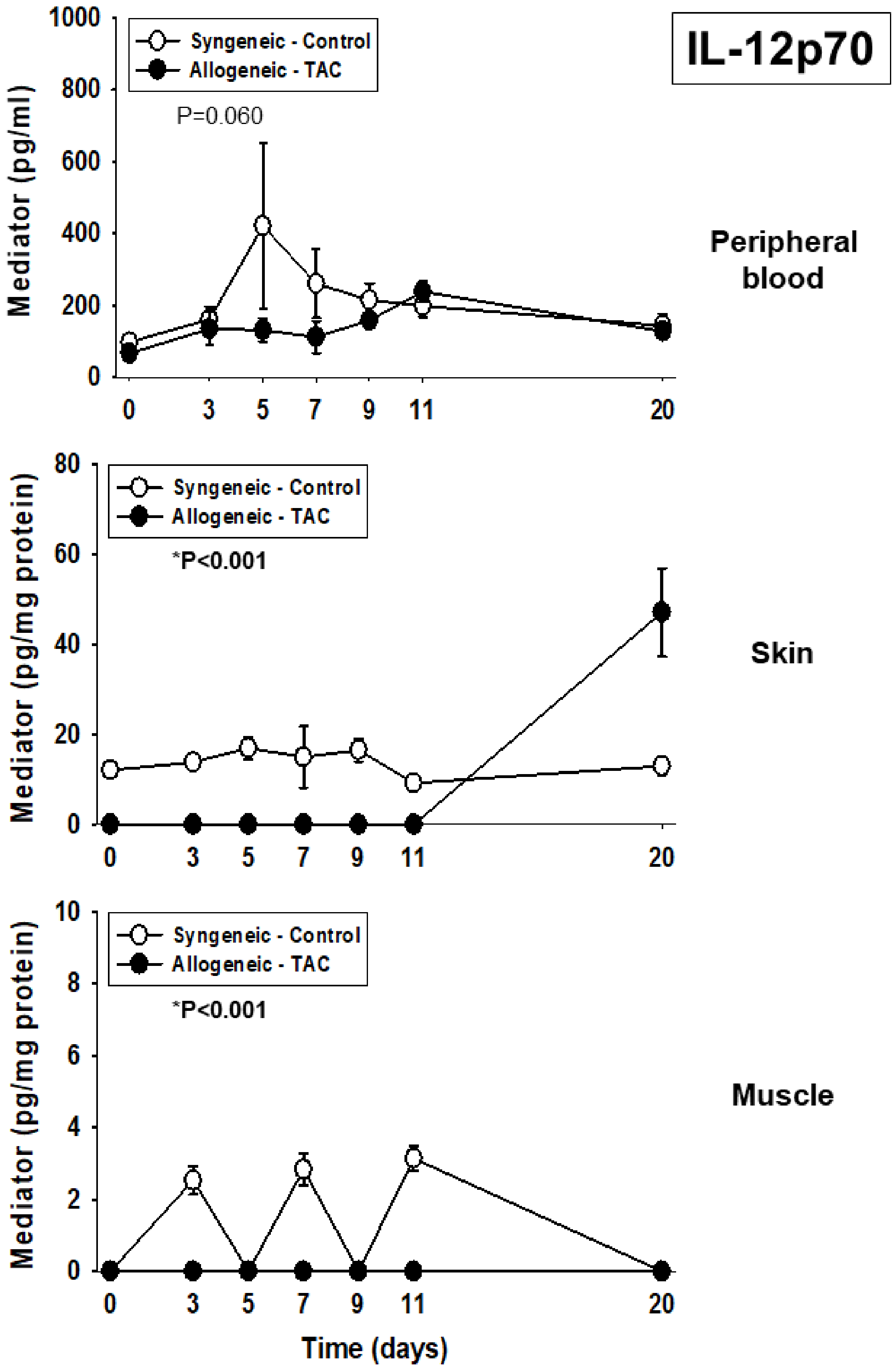
IL-12p70
Mediator (pg/ml)
1000
800
600
400
200
0
Syngeneic - Control
Allogeneic - TAC
P=0.060
0
3
5
7
9
11
20
Peripheral blood
Mediator (pg/mg protein)
80
60
40
20
0
Syngeneic - Control
Allogeneic - TAC
*P<0.001
0
3
5
7
9
11
20
Skin
Mediator (pg/mg protein)
10
8
6
4
2
0
Syngeneic - Control
Allogeneic - TAC
*P<0.001
0
3
5
7
9
11
20
Time (days)
Muscle

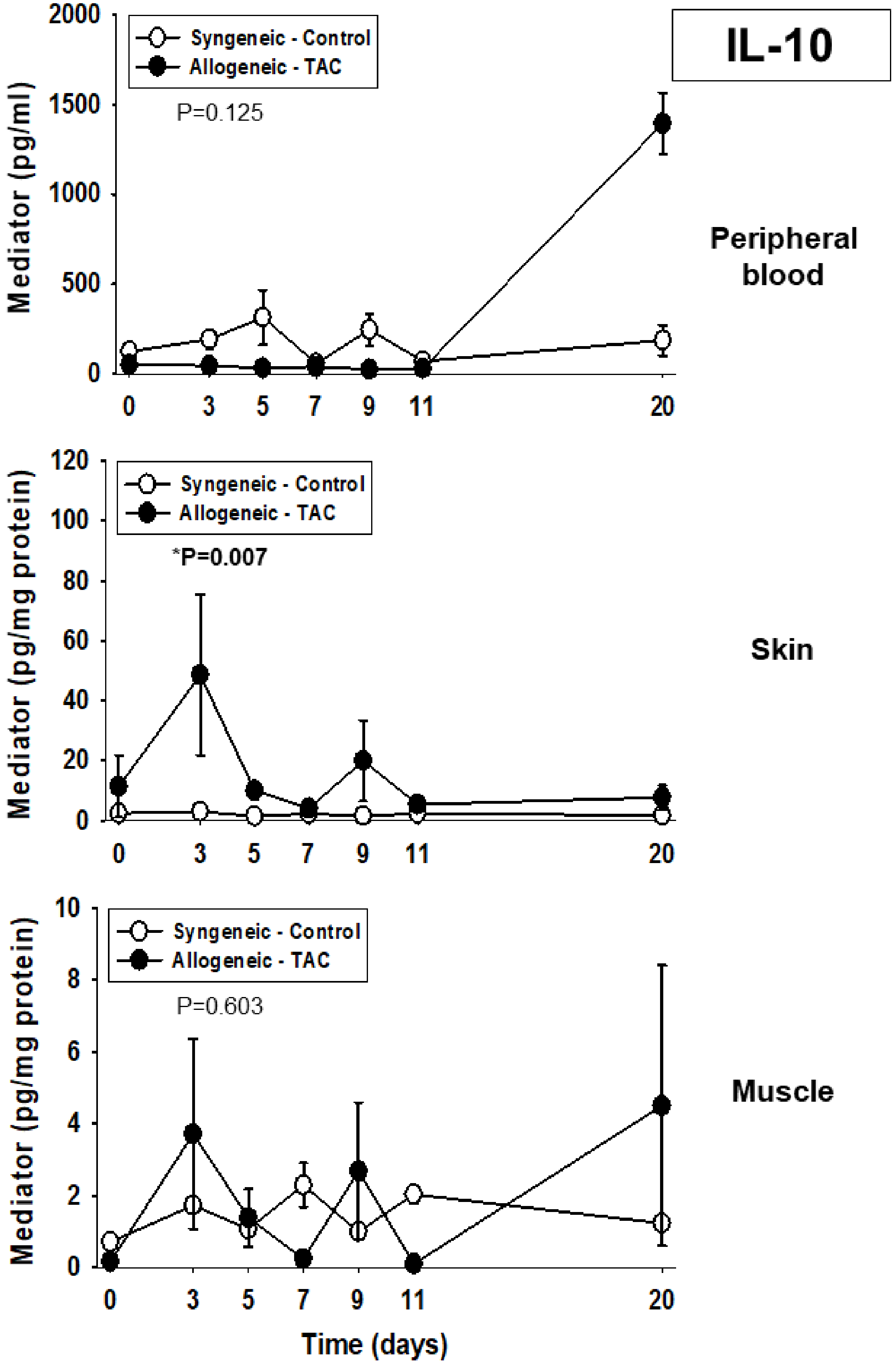
IL-10
Syngeneic - Control
Allogeneic - TAC
P=0.125
Mediator (pg/ml)
2000
1500
1000
500
0
0 3 5 7 9 11 20
Peripheral blood
Syngeneic - Control
Allogeneic - TAC
*P=0.007
Mediator (pg/mg protein)
120
100
80
60
40
20
0
0 3 5 7 9 11 20
Skin
Syngeneic - Control
Allogeneic - TAC
P=0.603
Mediator (pg/mg protein)
10
8
6
4
2
0
0 3 5 7 9 11 20
Time (days)
Muscle

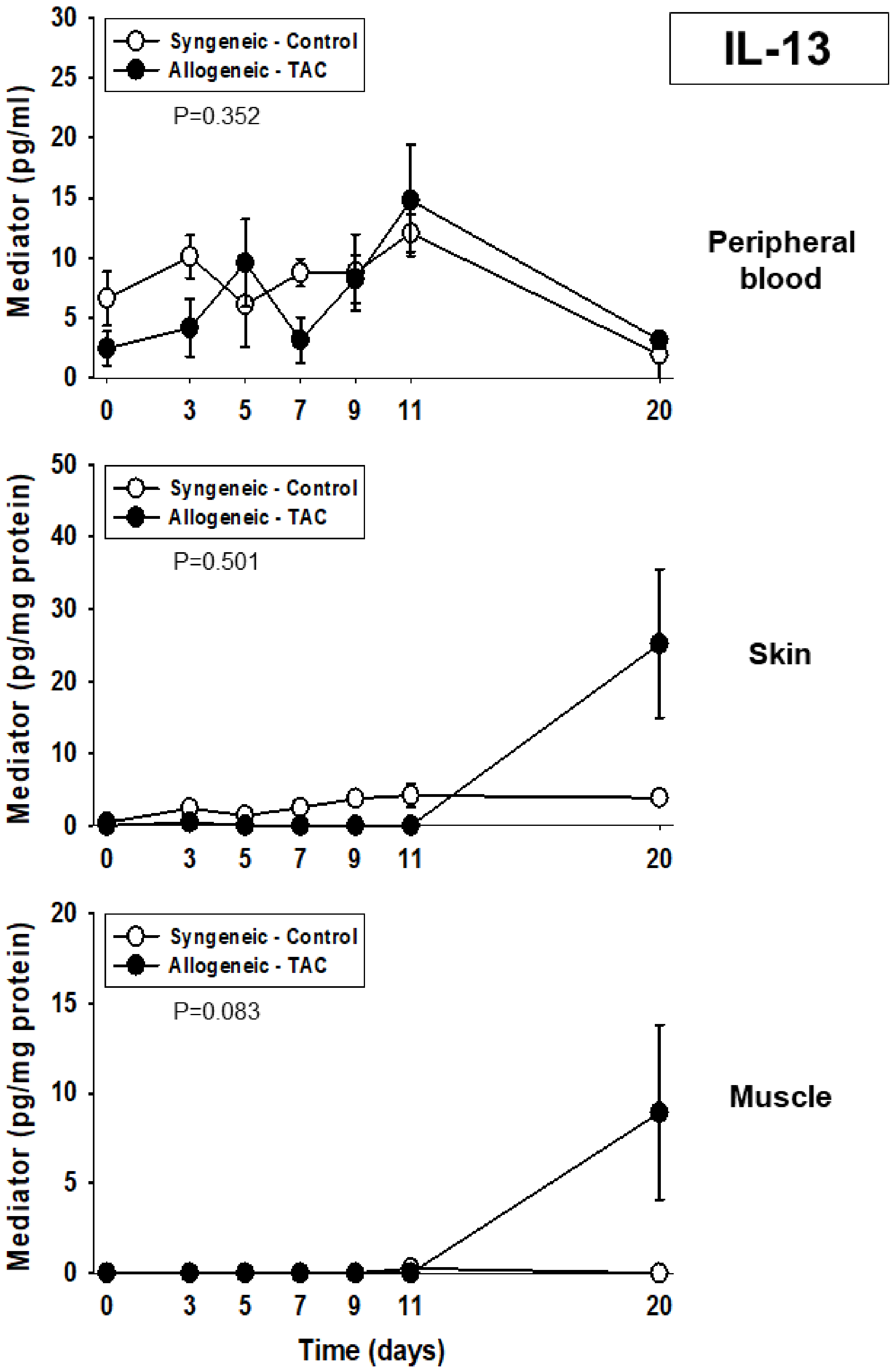
IL-13
Syngeneic - Control
Allogeneic - TAC
P=0.352
Mediator (pg/ml)
0
5
10
15
20
25
30
Peripheral blood
0
3
5
7
9
11
20
Syngeneic - Control
Allogeneic - TAC
P=0.501
Mediator (pg/mg protein)
0
10
20
30
40
50
Skin
0
3
5
7
9
11
20
Syngeneic - Control
Allogeneic - TAC
P=0.083
Mediator (pg/mg protein)
0
5
10
15
20
Muscle
0
3
5
7
9
11
20
Time (days)

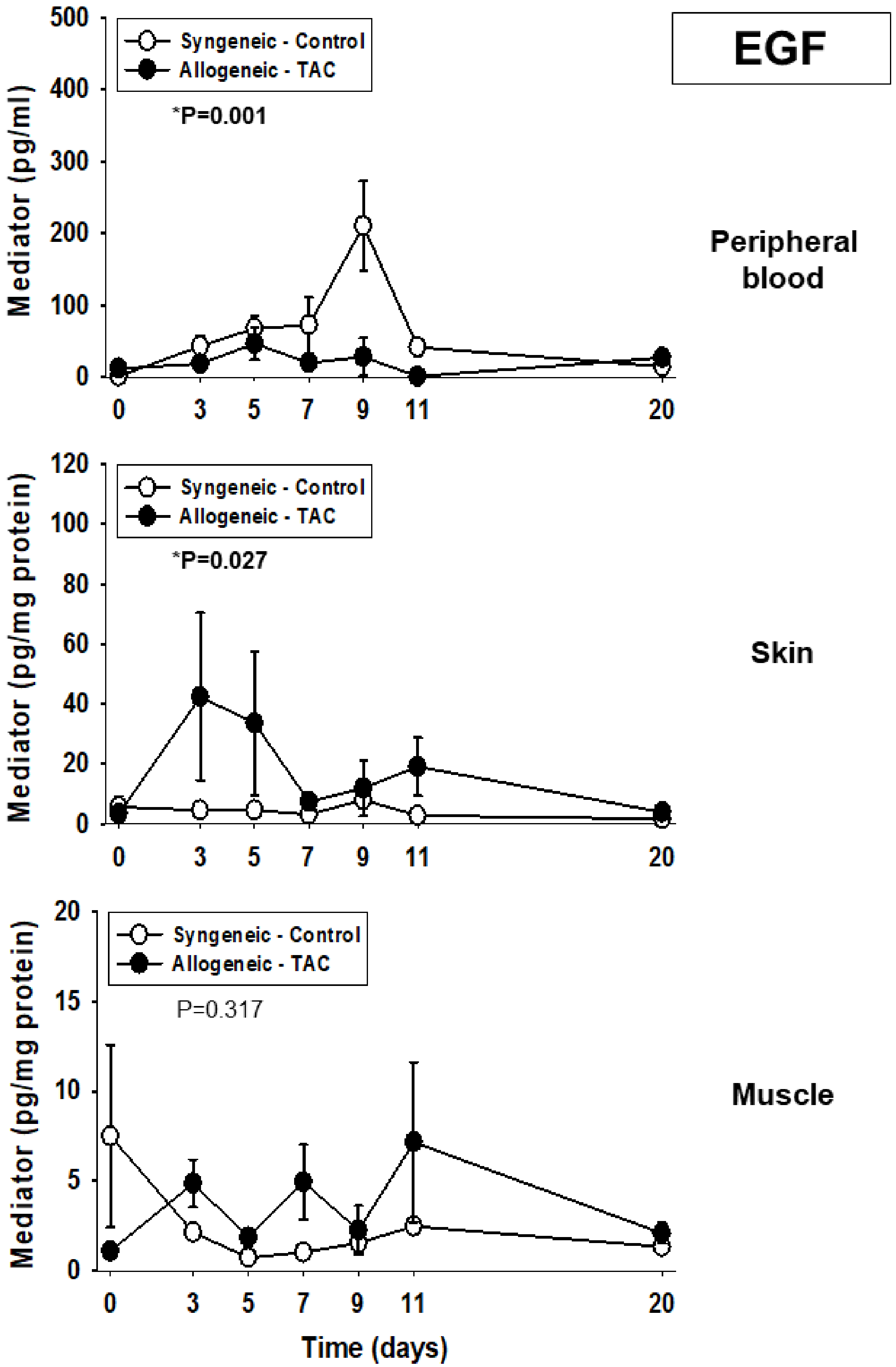
EGF
Syngeneic - Control
Allogeneic - TAC
*P=0.001
Mediator (pg/ml)
Peripheral blood
Syngeneic - Control
Allogeneic - TAC
*P=0.027
Mediator (pg/mg protein)
Skin
Syngeneic - Control
Allogeneic - TAC
P=0.317
Mediator (pg/mg protein)
Muscle
Time (days)

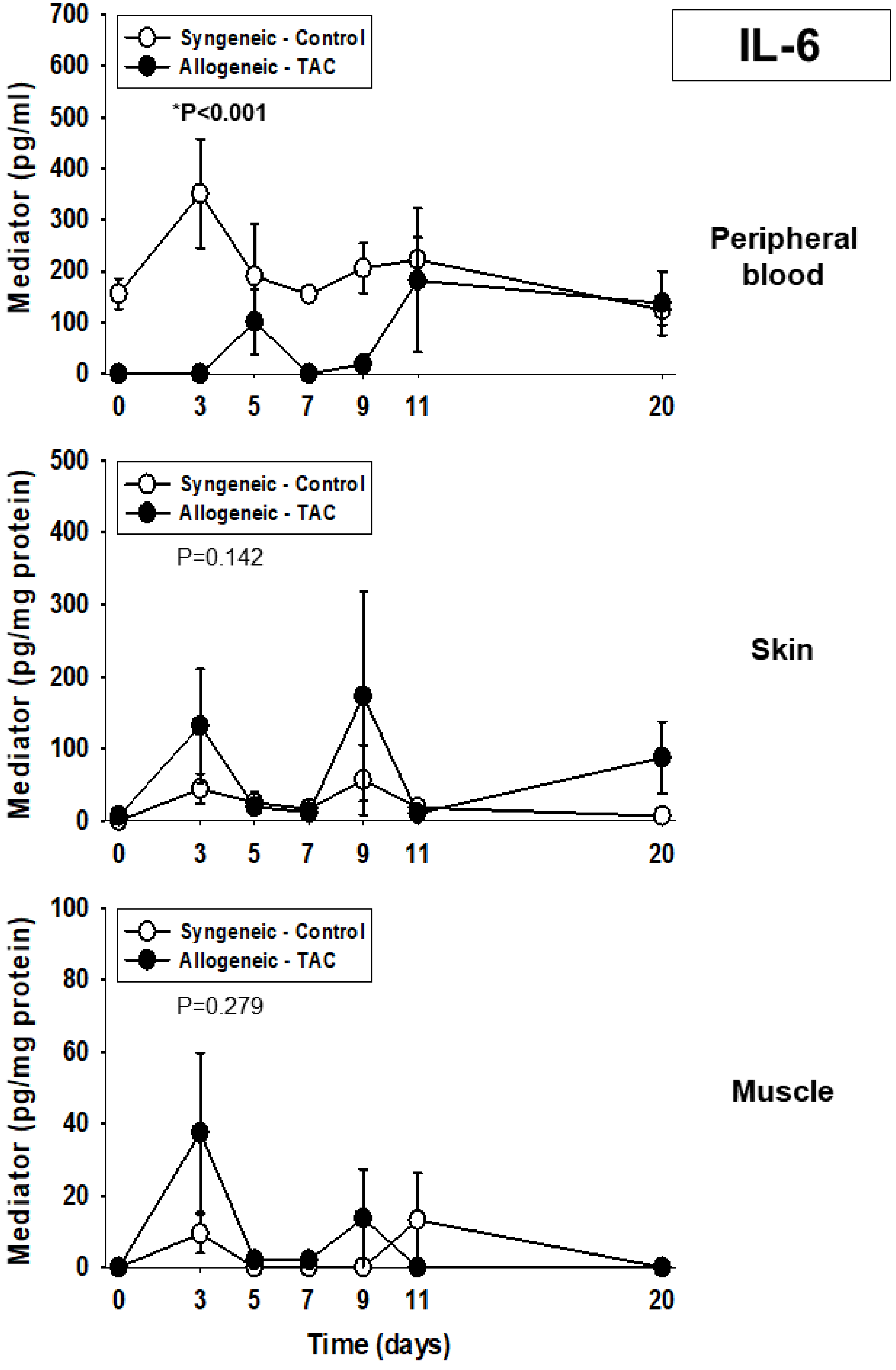
IL-6
Syngeneic - Control
Allogeneic - TAC
*P<0.001
Mediator (pg/ml)
700
600
500
400
300
200
100
0
0
3
5
7
9
11
20
Peripheral blood
Syngeneic - Control
Allogeneic - TAC
P=0.142
Mediator (pg/mg protein)
500
400
300
200
100
0
Skin
Syngeneic - Control
Allogeneic - TAC
P=0.279
Mediator (pg/mg protein)
100
80
60
40
20
0
Muscle
Time (days)

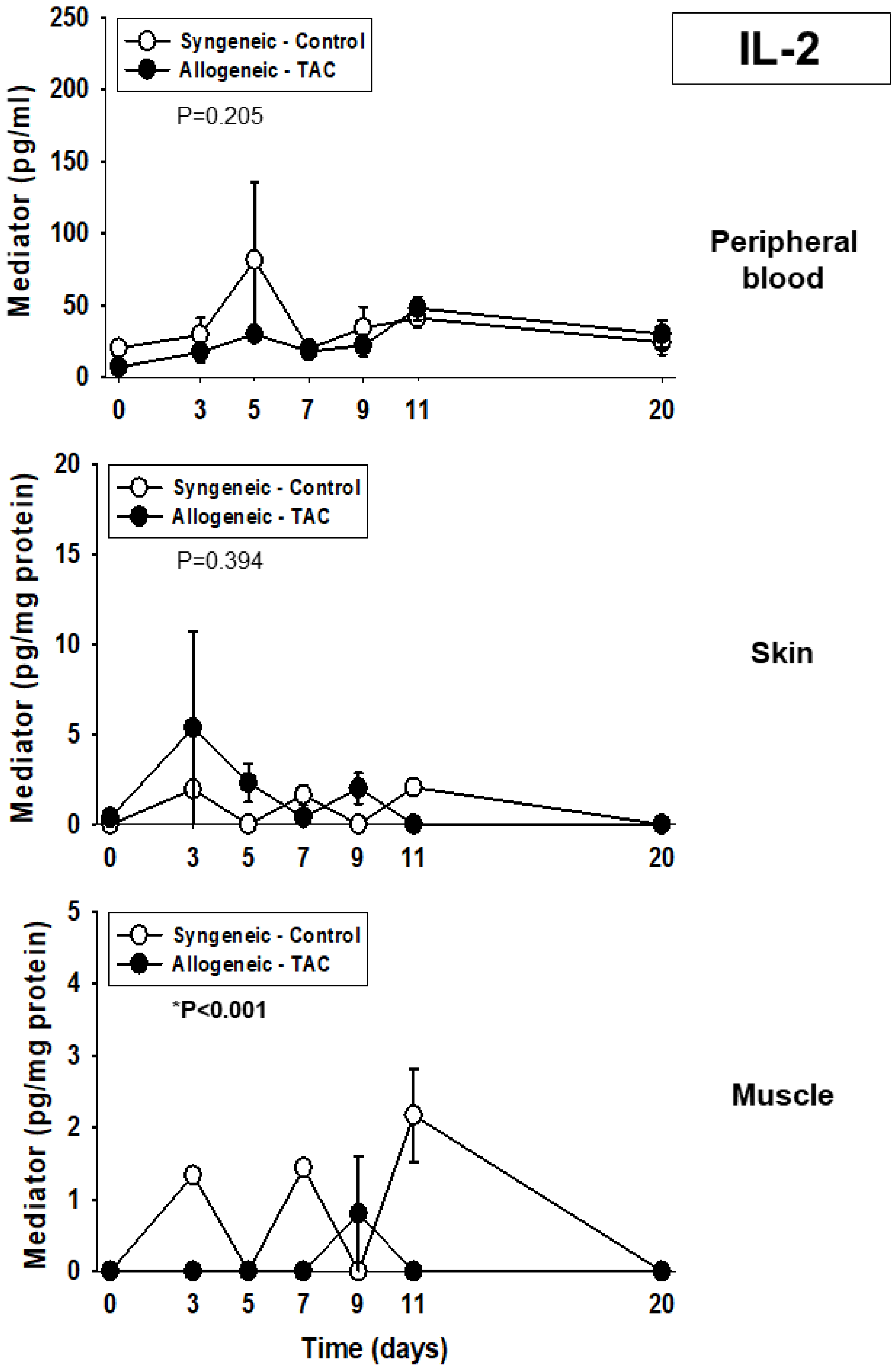
IL-2
Syngeneic - Control
Allogeneic - TAC
P=0.205
Mediator (pg/ml)
250
200
150
100
50
0
0 3 5 7 9 11 20
Peripheral blood
Syngeneic - Control
Allogeneic - TAC
P=0.394
Mediator (pg/mg protein)
20
15
10
5
0
0 3 5 7 9 11 20
Skin
Syngeneic - Control
Allogeneic - TAC
*P<0.001
Mediator (pg/mg protein)
5
4
3
2
1
0
0 3 5 7 9 11 20
Time (days)
Muscle

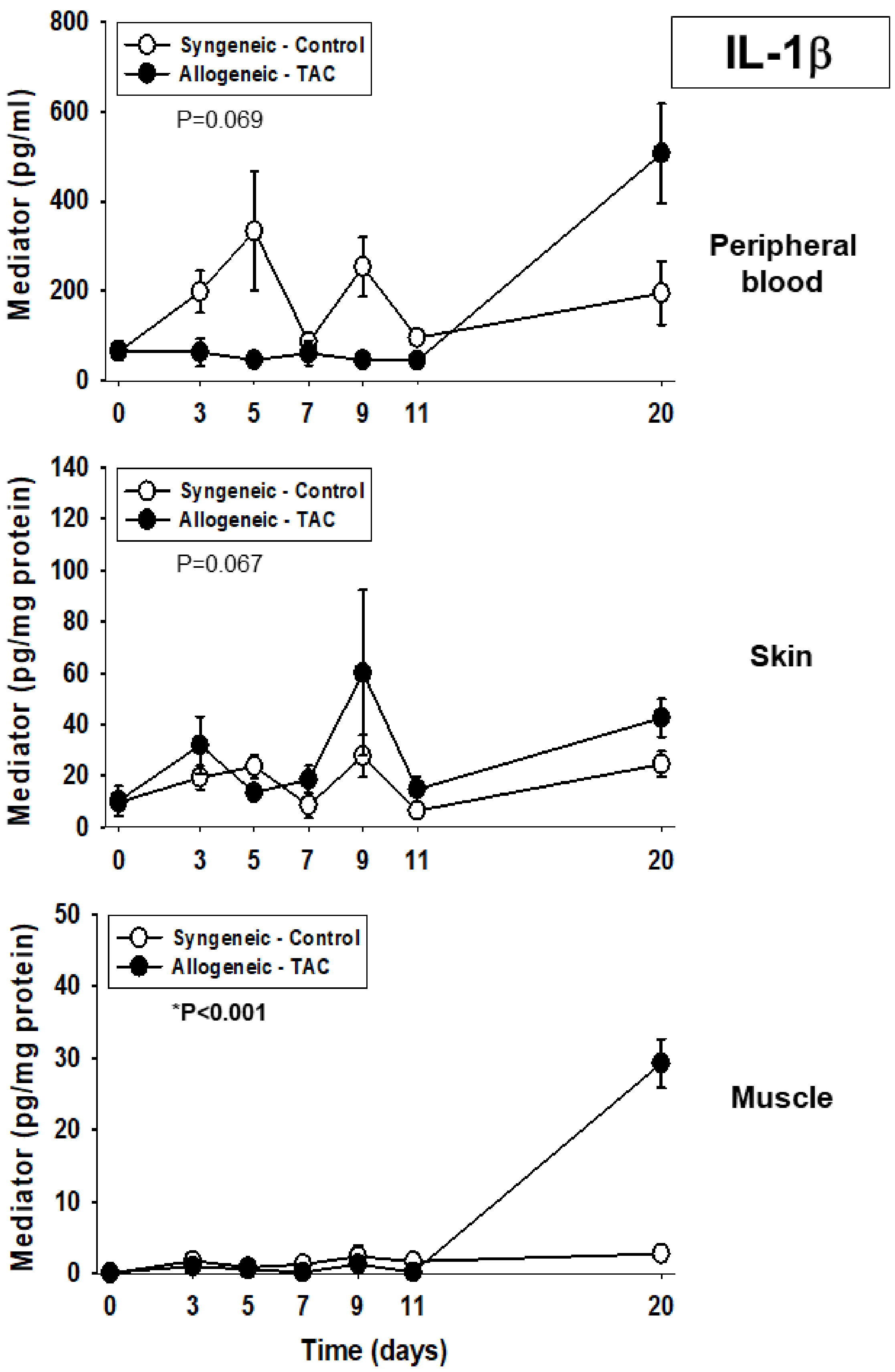
IL-1β
Syngeneic - Control
Allogeneic - TAC
P=0.069
Mediator (pg/ml)
800
600
400
200
0
0
3
5
7
9
11
20
Peripheral blood
Syngeneic - Control
Allogeneic - TAC
P=0.067
Mediator (pg/mg protein)
140
120
100
80
60
40
20
0
0
3
5
7
9
11
20
Skin
Syngeneic - Control
Allogeneic - TAC
*P<0.001
Mediator (pg/mg protein)
50
40
30
20
10
0
0
3
5
7
9
11
20
Time (days)
Muscle

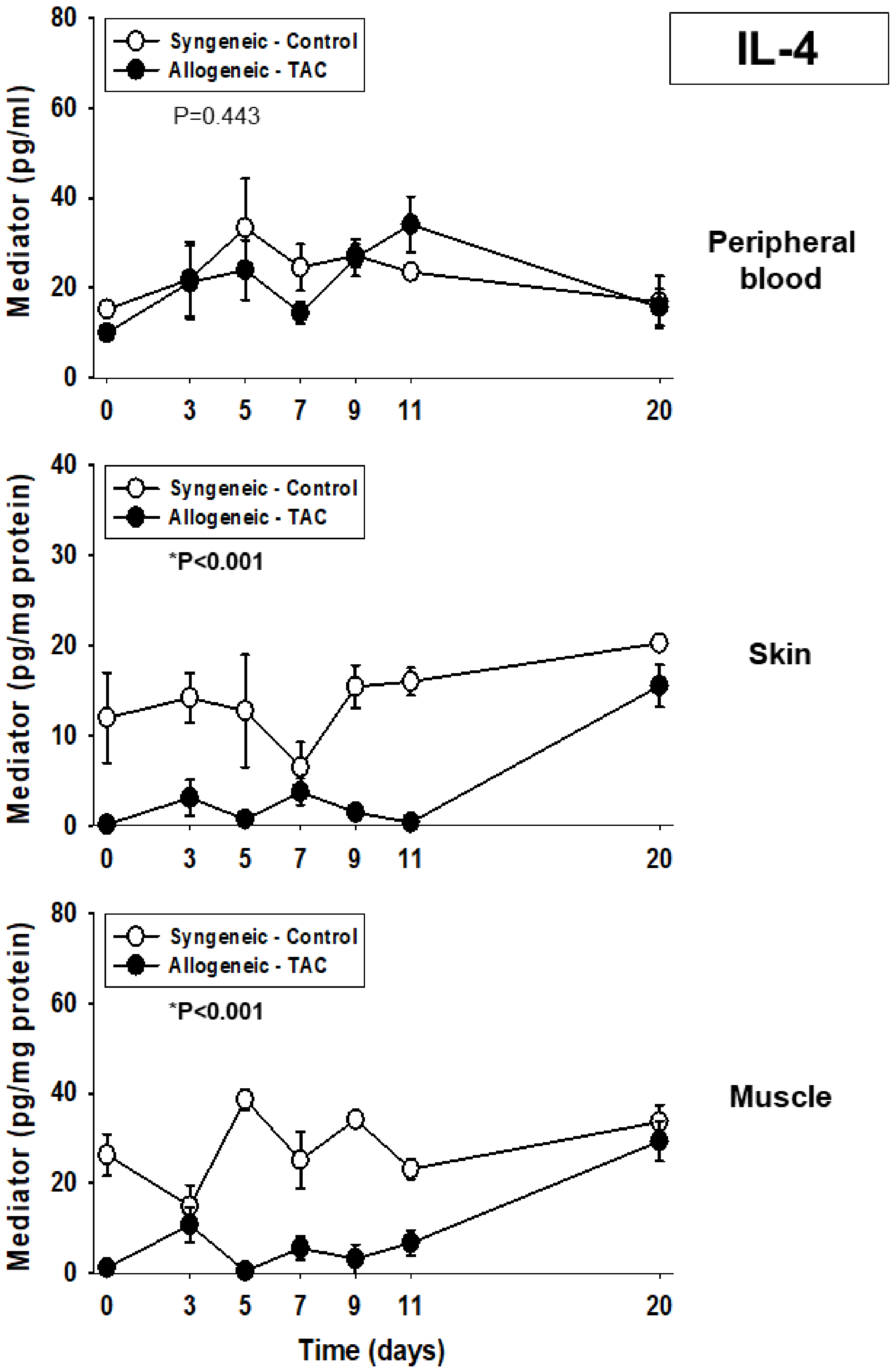
IL-4
Syngeneic - Control
Allogeneic - TAC
P=0.443
Peripheral blood
Mediator (pg/ml)
80
60
40
20
0
0 3 5 7 9 11 20
Syngeneic - Control
Allogeneic - TAC
*P<0.001
Skin
Mediator (pg/mg protein)
40
30
20
10
0
0 3 5 7 9 11 20
Syngeneic - Control
Allogeneic - TAC
*P<0.001
Muscle
Mediator (pg/mg protein)
80
60
40
20
0
0 3 5 7 9 11 20
Time (days)

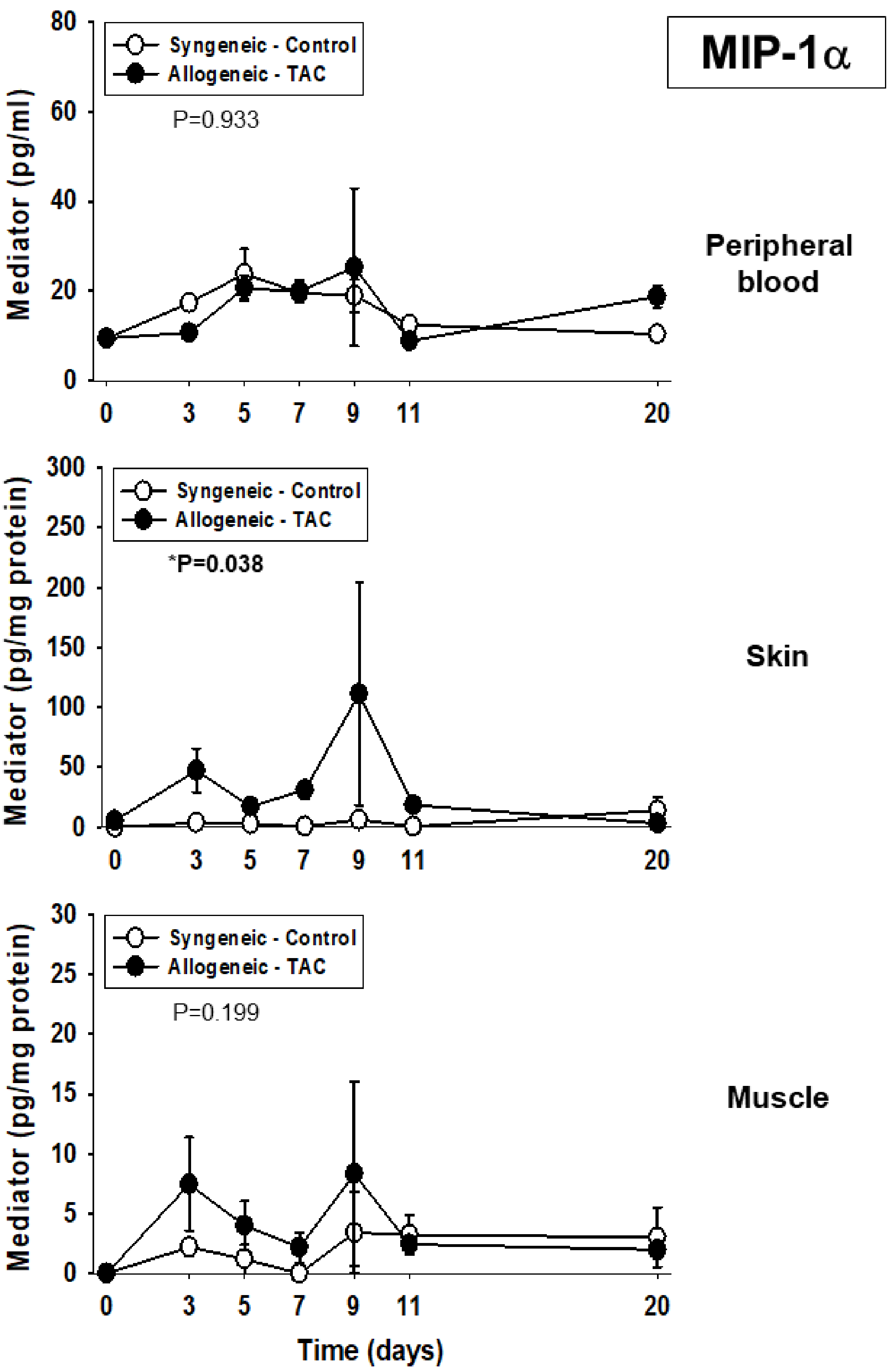
MIP-1α
Syngeneic - Control
Allogeneic - TAC
P=0.933
Mediator (pg/ml)
0
20
40
60
80
0 3 5 7 9 11 20
Peripheral blood
Syngeneic - Control
Allogeneic - TAC
*P=0.038
Mediator (pg/mg protein)
0
50
100
150
200
250
300
0 3 5 7 9 11 20
Skin
Syngeneic - Control
Allogeneic - TAC
P=0.199
Mediator (pg/mg protein)
0
5
10
15
20
25
30
0 3 5 7 9 11 20
Time (days)
Muscle

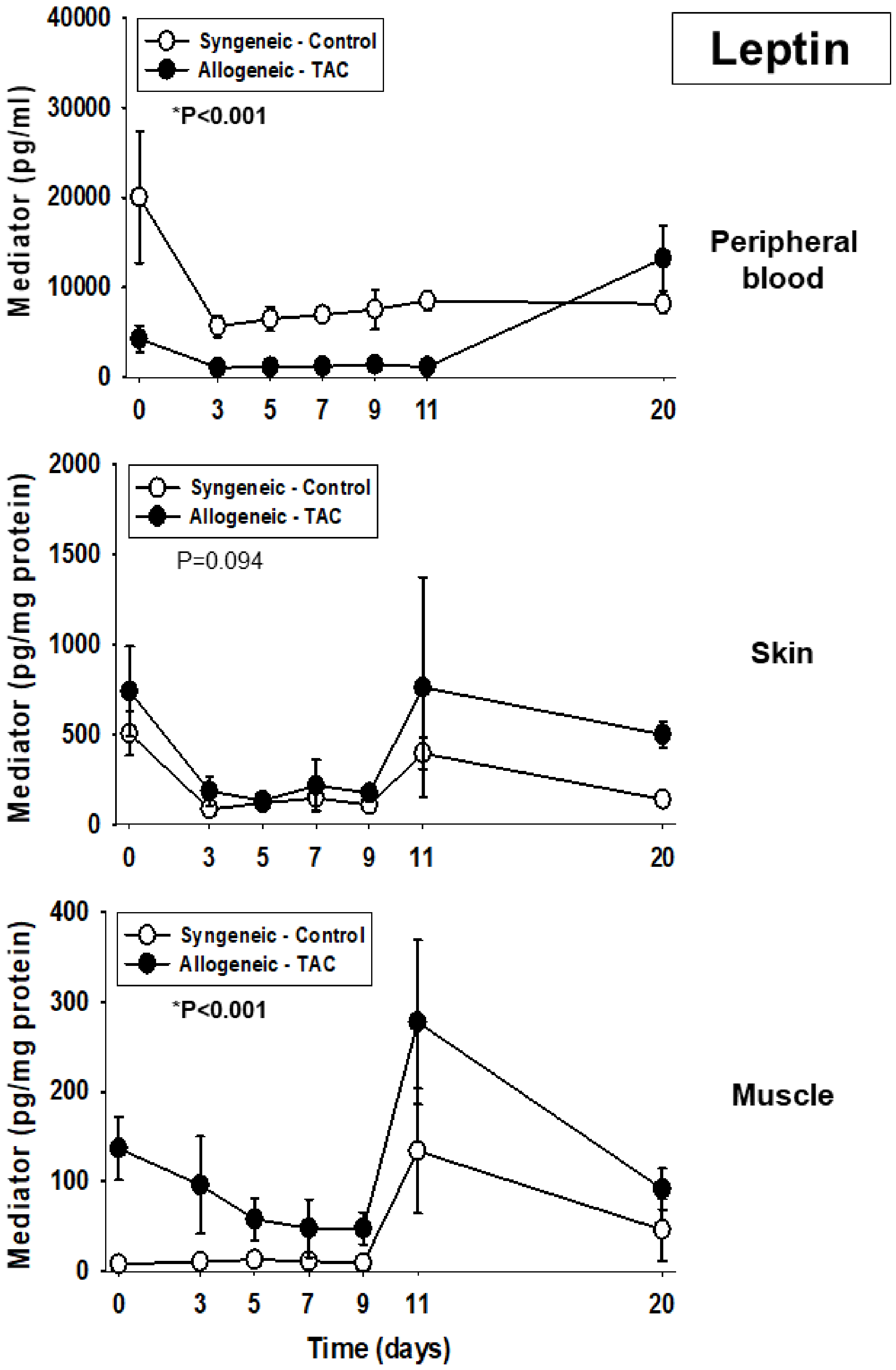
Leptin
Syngeneic - Control
Allogeneic - TAC
*P<0.001
Mediator (pg/ml)
40000
30000
20000
10000
0
0
3
5
7
9
11
20
Peripheral blood
Syngeneic - Control
Allogeneic - TAC
P=0.094
Mediator (pg/mg protein)
2000
1500
1000
500
0
0
3
5
7
9
11
20
Skin
Syngeneic - Control
Allogeneic - TAC
*P<0.001
Mediator (pg/mg protein)
400
300
200
100
0
0
3
5
7
9
11
20
Time (days)
Muscle

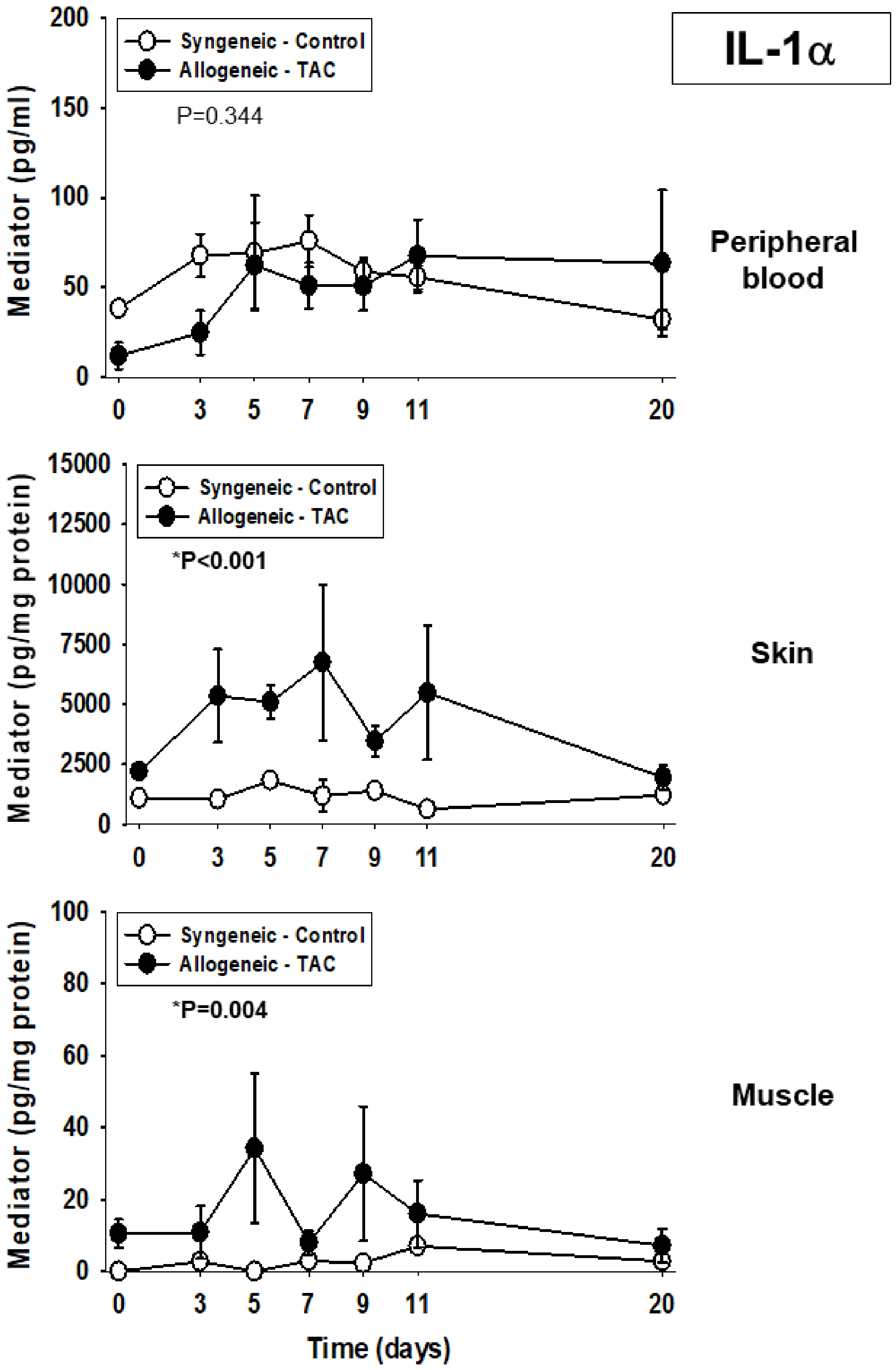
IL-1α
Syngeneic - Control
Allogeneic - TAC
P=0.344
Mediator (pg/ml)
200
150
100
50
0
0
3
5
7
9
11
20
Peripheral blood
Syngeneic - Control
Allogeneic - TAC
*P<0.001
Mediator (pg/mg protein)
15000
12500
10000
7500
5000
2500
0
0
3
5
7
9
11
20
Skin
Syngeneic - Control
Allogeneic - TAC
*P=0.004
Mediator (pg/mg protein)
100
80
60
40
20
0
0
3
5
7
9
11
20
Time (days)
Muscle

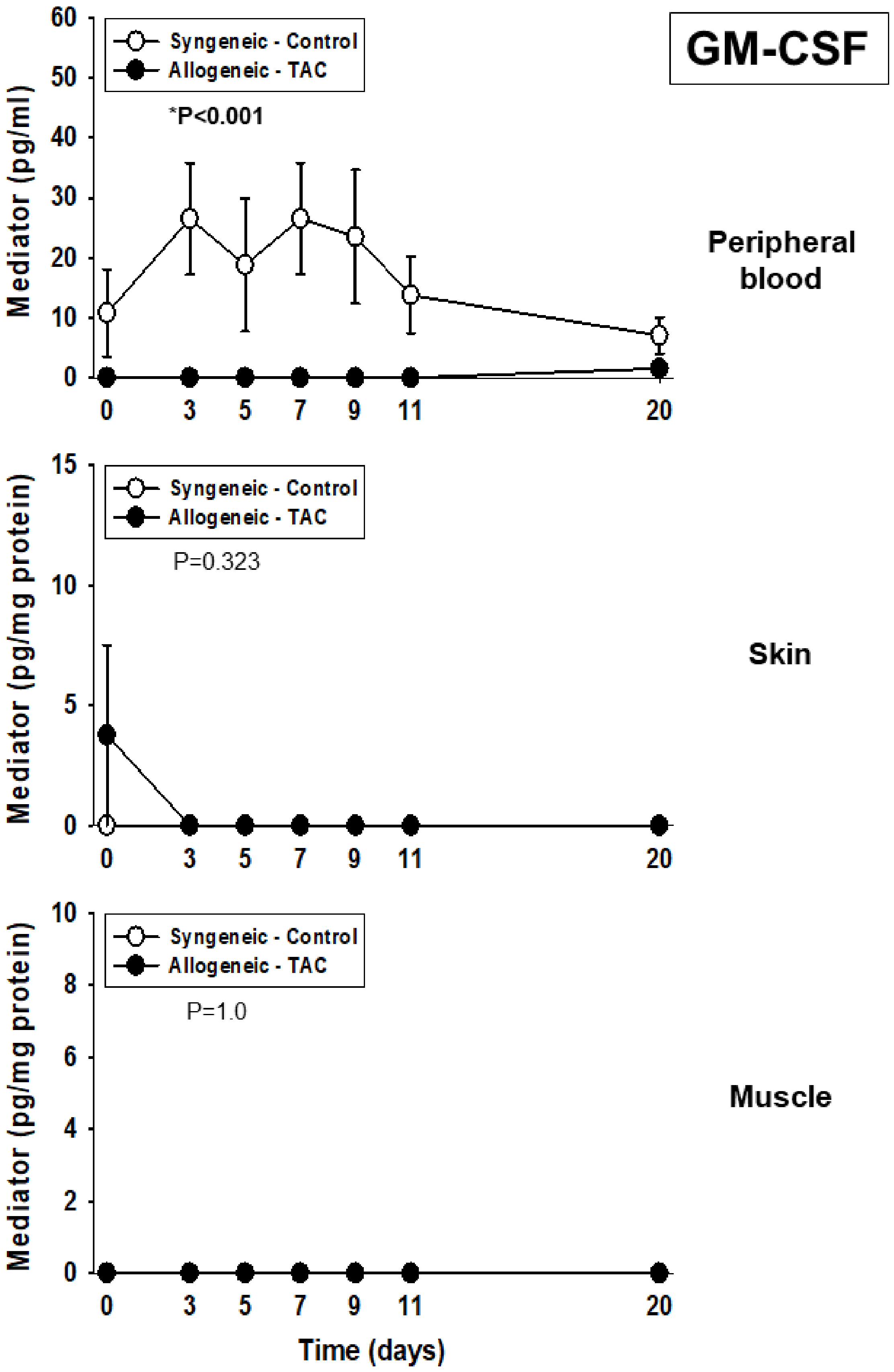
GM-CSF
Syngeneic - Control
Allogeneic - TAC
*P<0.001
Mediator (pg/ml)
Peripheral blood
P=0.323
Mediator (pg/mg protein)
Skin
P=1.0
Mediator (pg/mg protein)
Muscle
Time (days)

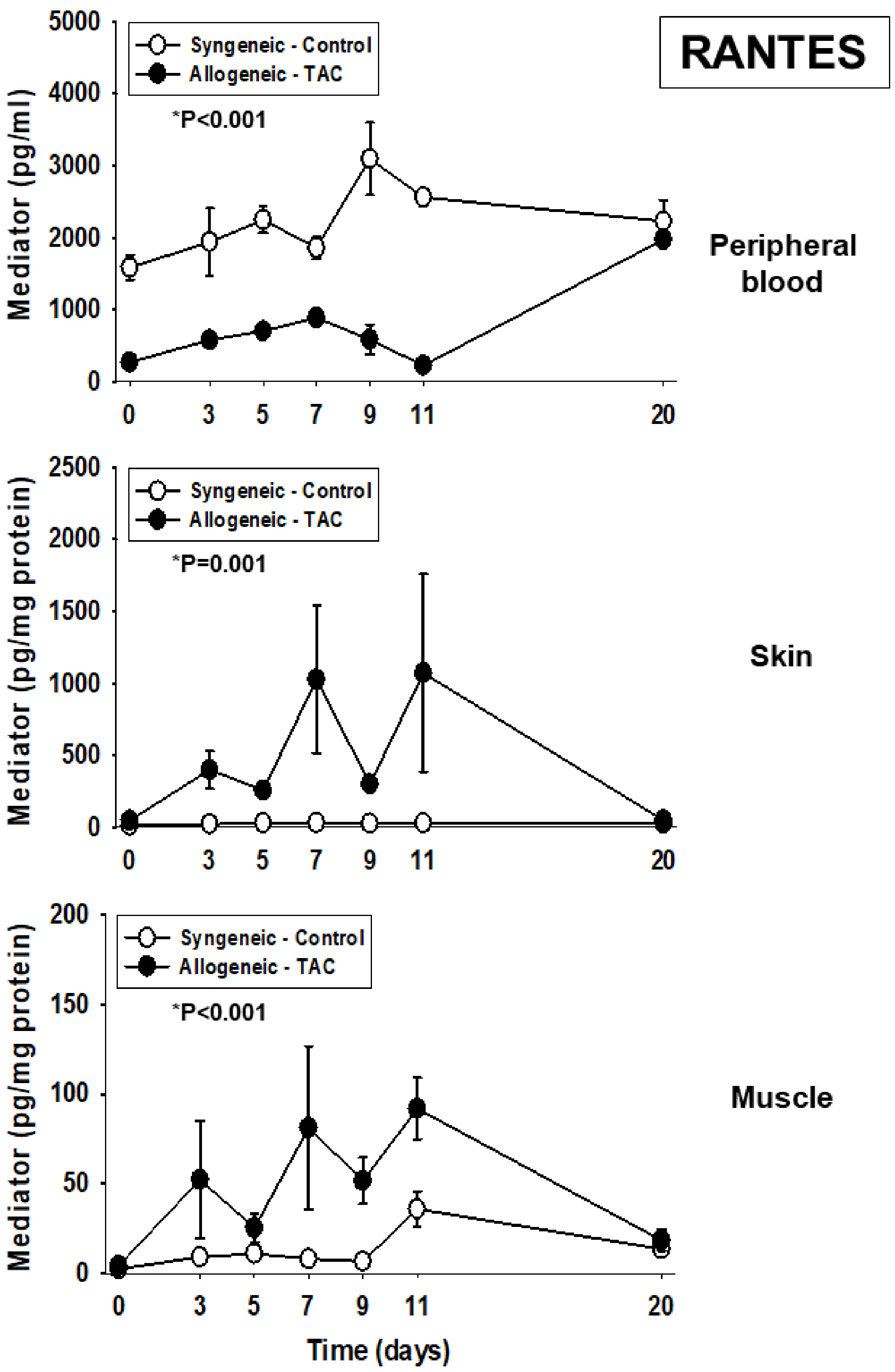
RANTES
Syngeneic - Control
Allogeneic - TAC
*P<0.001
Peripheral blood
Mediator (pg/ml)
5000
4000
3000
2000
1000
0
0
3
5
7
9
11
20
Syngeneic - Control
Allogeneic - TAC
*P=0.001
Skin
Mediator (pg/mg protein)
2500
2000
1500
1000
500
0
Syngeneic - Control
Allogeneic - TAC
*P<0.001
Muscle
Mediator (pg/mg protein)
200
150
100
50
0
Time (days)

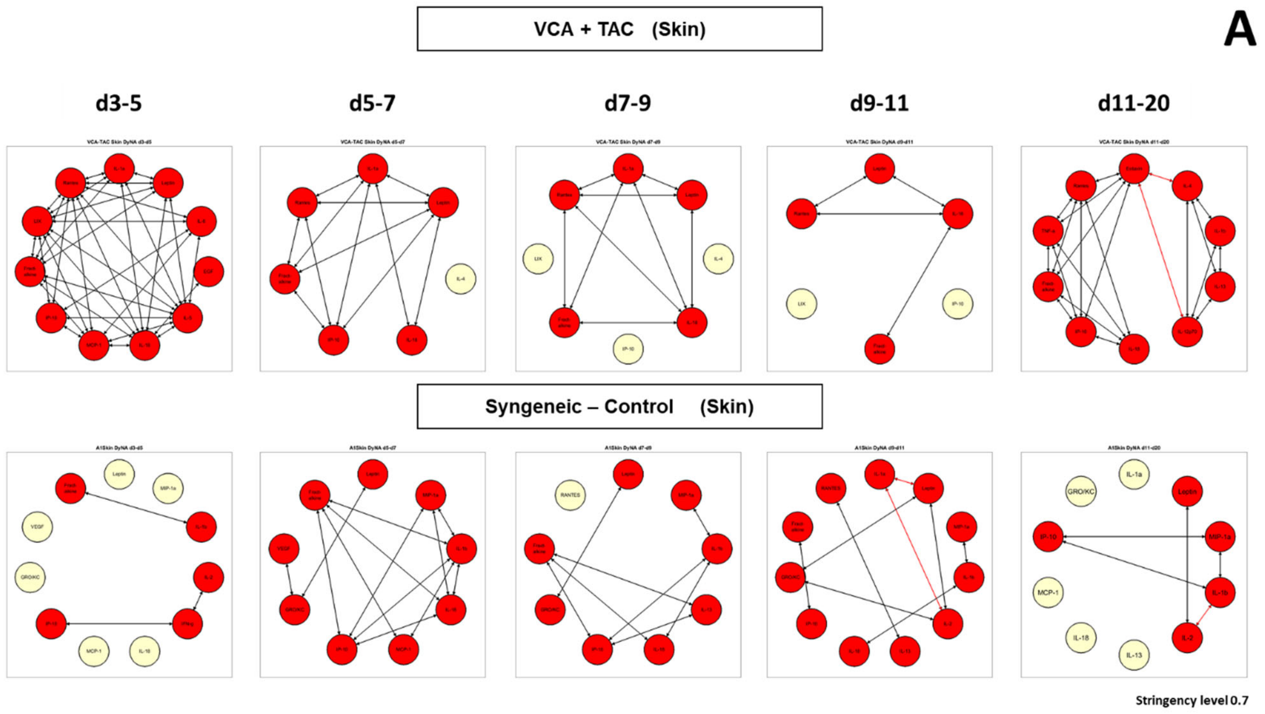
VCA + TAC (Skin)
A
d3-5
d5-7
d7-9
d9-11
d11-20
Syngeneic – Control (Skin)
Stringency level 0.7

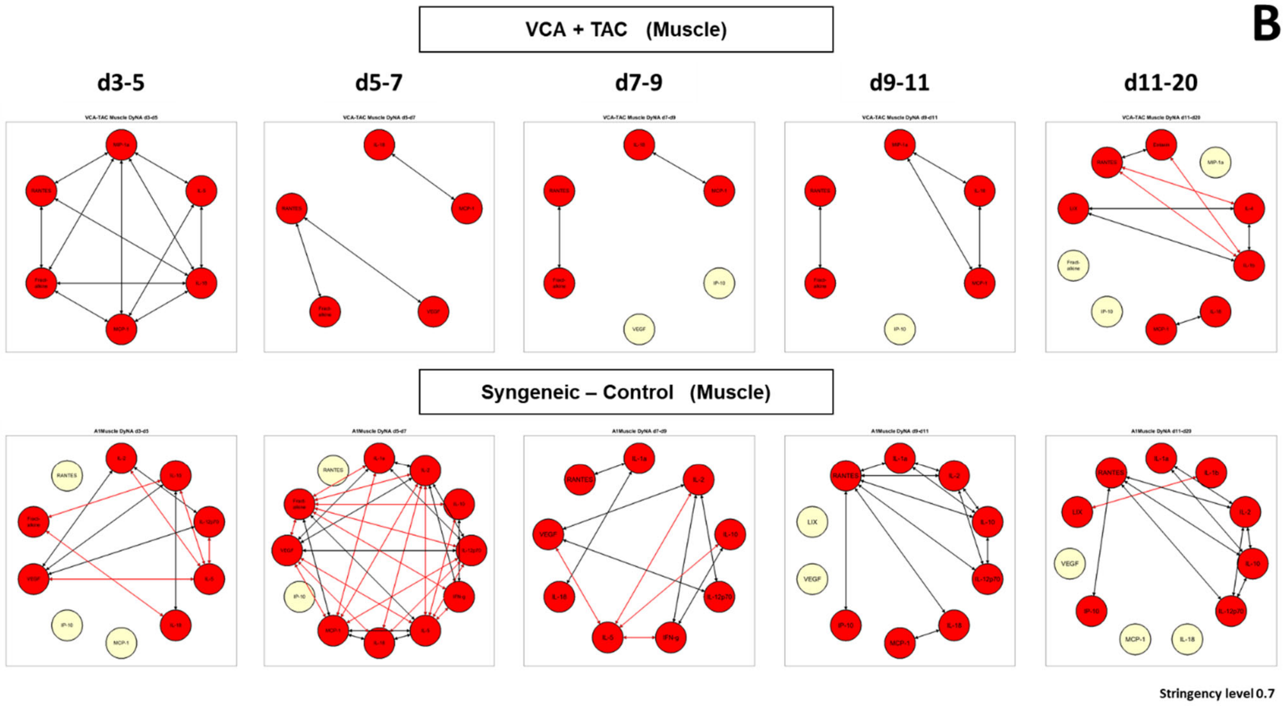
VCA + TAC (Muscle)
B
d3-5
d5-7
d7-9
d9-11
d11-20
Syngeneic – Control (Muscle)
Stringency level 0.7

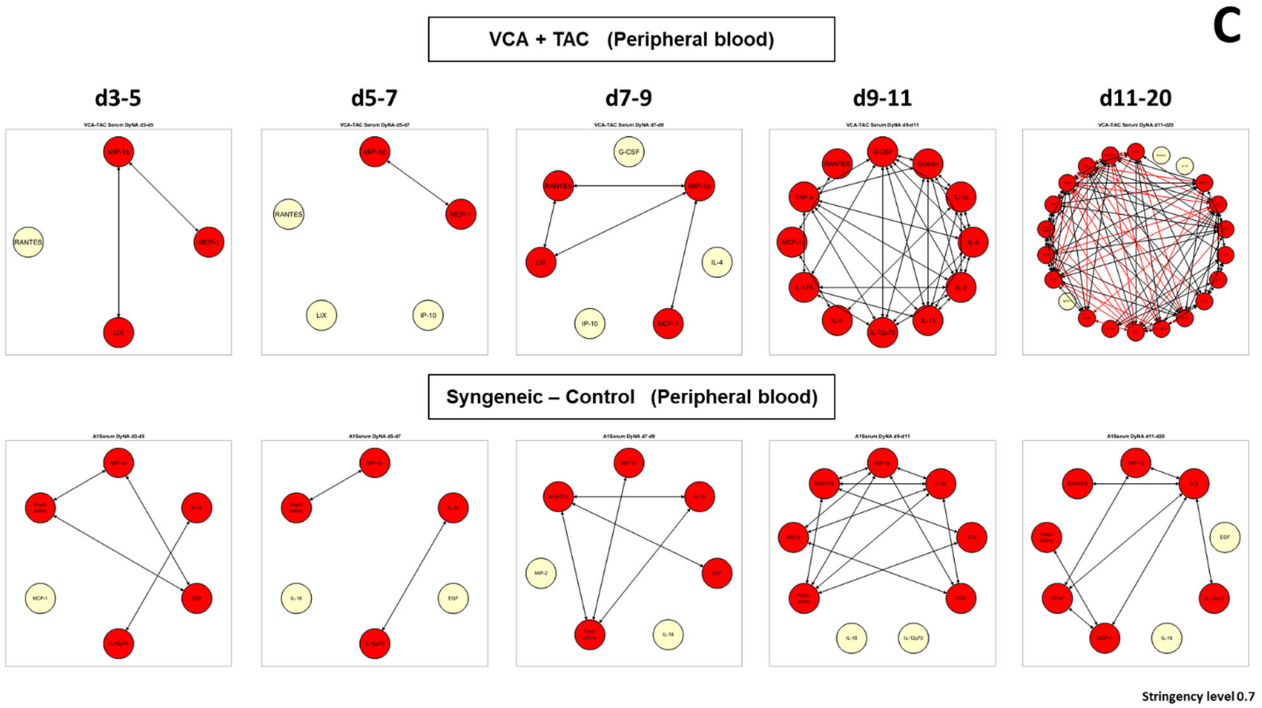

Supplementary Fig. 3. Dynamic inflammatory networks in skin, muscle and peripheral blood in rats undergoing VCA +TAC vs. Syngeneic Control. LEW rat recipients received full MHC-mismatched BN limbs with TAC (1 mg/kg/day, i.p.) until postoperative day 20 followed by drug withdrawal as described in *Materials and Methods*. LEW rat recipients that received MHC-matched LEW limbs without TAC served as control. Peripheral blood, skin and muscle tissue samples were collected at 0, 3, 5, 7, 9, 11, and 20 days and assayed for 27 inflammatory mediators using the rat multiplex Luminex™ assay followed by DyNA performed during five time-intervals as described in *Materials and Methods.* Figure shows the individual networks (stringency level 0.7) in VCA + TAC vs. Syngeneic-Control in skin (Panel **A**), muscle (Panel **B**) and peripheral blood (Panel **C**) as indicated.

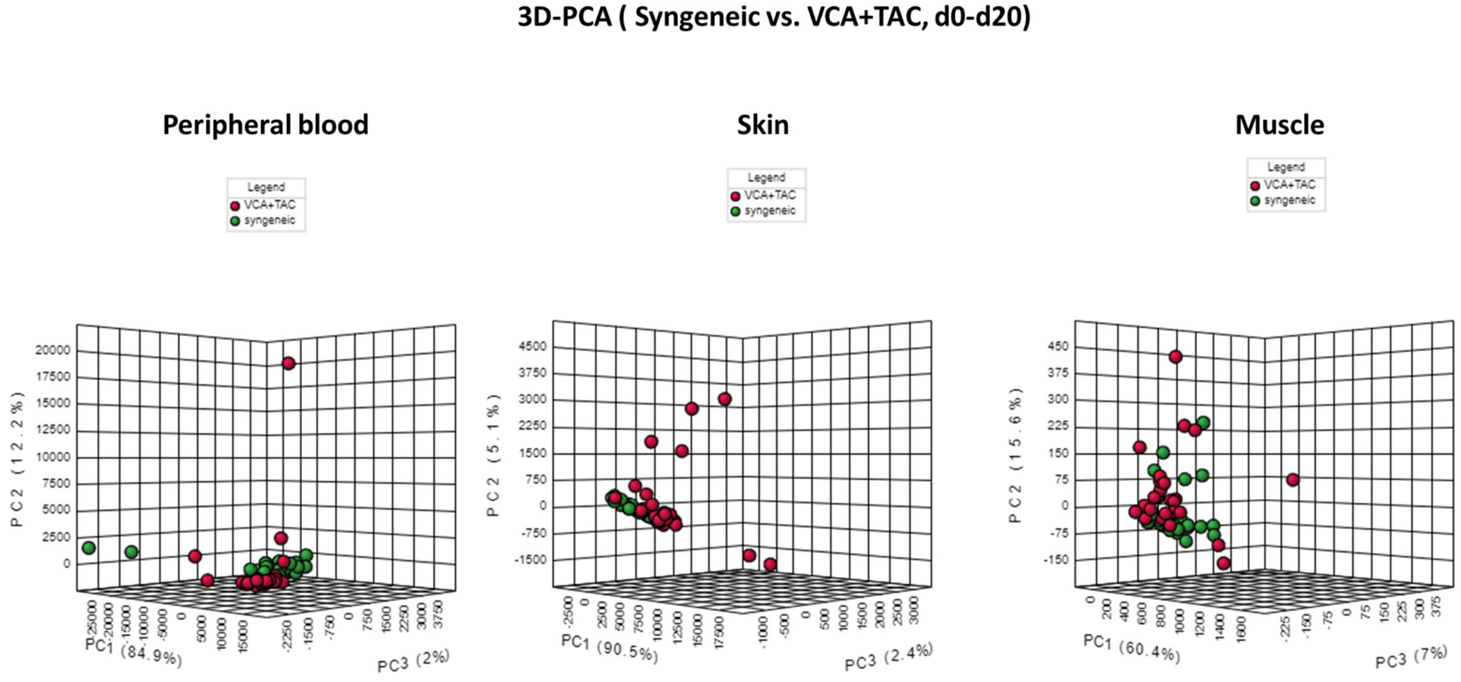

Supplementary Figure 4. Principal Component Analysis (PCA) in VCA + TAC vs. Syngeneic-Control. LEW rat recipients received full MHC-mismatched BN limbs with TAC (1 mg/kg/day, i.p.) until postoperative day 20 followed by drug withdrawal as described in *Materials and Methods*. LEW rat recipients that received MHC-matched LEW limbs without TAC served as control. Peripheral blood, skin and muscle tissue samples were collected at 0, 3, 5, 7, 9, 11, and 20 days and assayed for 27 inflammatory mediators using the rat multiplex Luminex™ assay followed by PCA performed as described in *Materials and Methods.* Figure shows the 3D-PCA results for the first three components in VCA + TAC (red symbols) vs. Syngeneic-Control (green symbols) in peripheral blood, skin, and muscle as indicated.

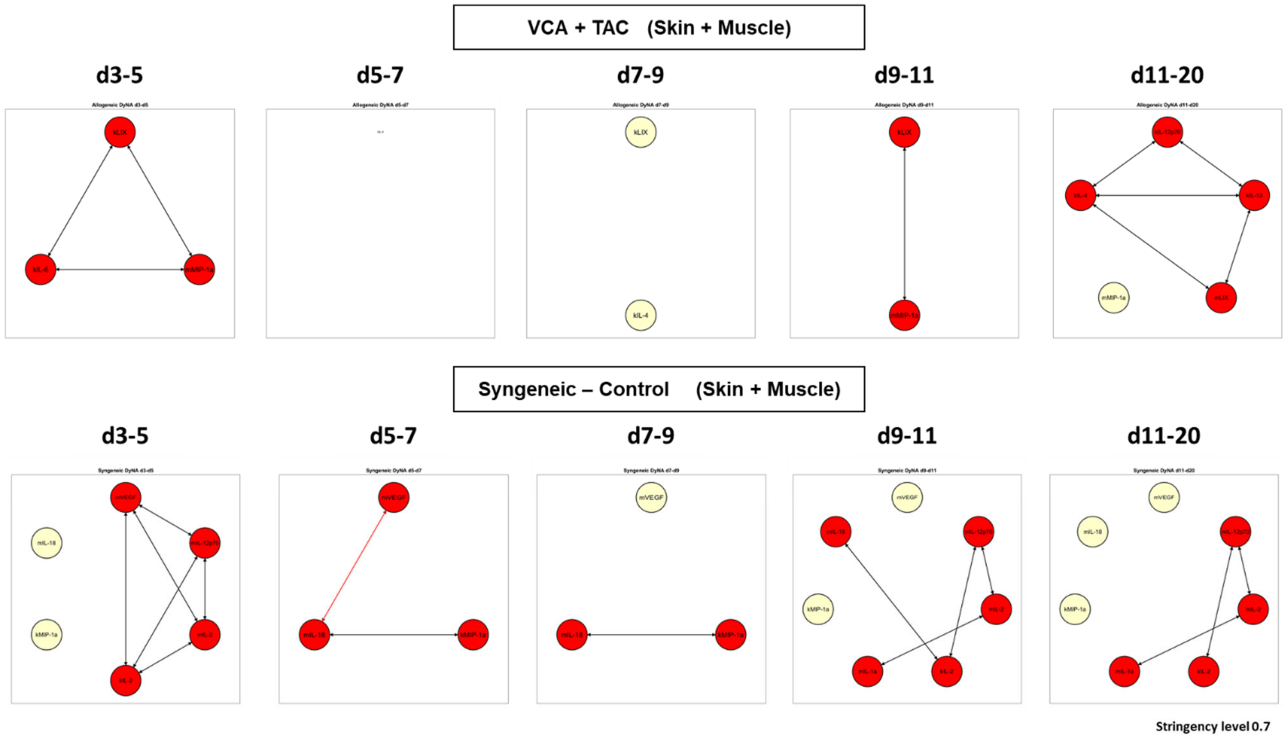

Supplementary Figure 5. Dynamic inflammatory networks in skin and muscle (combined) in rats undergoing VCA +TAC vs. Syngeneic Control. LEW rat recipients received full MHC-mismatched BN limbs with TAC (1 mg/kg/day, i.p.) until postoperative day 20 followed by drug withdrawal as described in *Materials and Methods*. LEW rat recipients that received MHC-matched LEW limbs without TAC served as control. Skin and muscle tissue samples were collected at 0, 3, 5, 7, 9, 11, and 20 days and assayed for 27 inflammatory mediators using the rat multiplex Luminex™ assay followed by DyNA performed during five time-intervals as described in *Materials and Methods*. Figure shows the individual networks (stringency level 0.7) in skin and muscle (combined) in VCA + TAC (Panel **A**) vs. Syngeneic-Control (Panel **B**) as indicated.

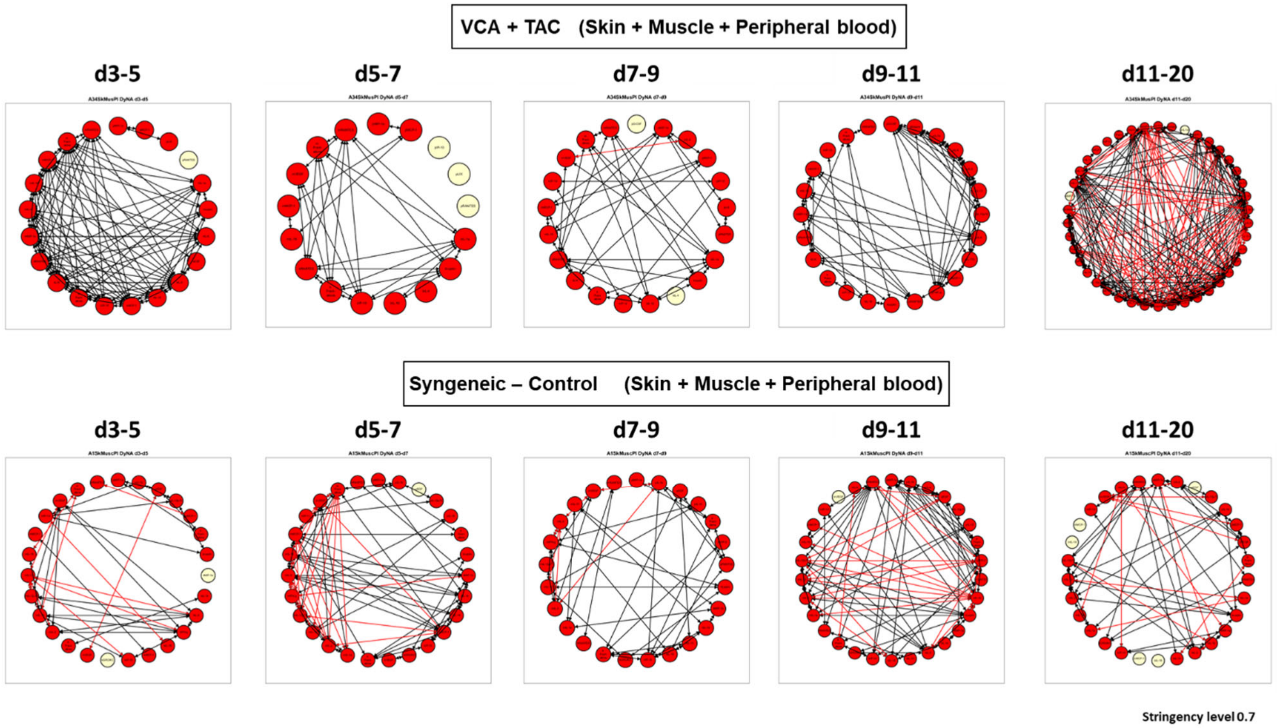

Supplementary Figure 6. Differential inflammatory network patterns in rats undergoing VCA +TAC vs. Syngeneic Control. LEW rat recipients received full MHC-mismatched BN limbs with TAC (1 mg/kg/day, i.p.) until postoperative day 20 followed by drug withdrawal as described in *Materials and Methods*. LEW rat recipients that received MHC-matched LEW limbs without TAC served as control. Peripheral blood, skin and muscle tissue samples were collected at 0, 3, 5, 7, 9, 11, and 20 days and assayed for 27 inflammatory mediators using the rat multiplex Luminex™ assay followed by DyNA as described in *Materials and Methods*. Figure shows the individual networks (stringency level 0.7) for skin, muscle and peripheral blood (combined) in VCA + TAC and Syngeneic-Control as indicated.

Supplementary Table 1. One-Way ANOVA of time-courses in Skin, Muscle and Peripheral blood of animals receiving TAC vs. Syngeneic Control.

Suppl. Table 1

P values (One-One-Way ANOVA or Kruskall-Wallis ANOVA on Ranks)

VCA + TAC

Mediator	Skin	Muscle	P. blood
G-CSF	0.095	0.07	0.033
Eotaxin	0.015	0.021	0.001
GM-CSF	0.423	1	0.052
IL-1α	0.14	0.858	0.203
Leptin	0.068	0.025	0.017
MIP-1α	0.018	0.2	0.015
IL-4	0.005	<0.001	0.056
IL-1β	0.072	0.038	0.106
IL-2	0.108	0.423	0.009
IL-6	0.042	0.13	0.034
EGF	0.082	0.078	0.061
IL-13	<0.001	0.004	0.031
IL-10	0.135	0.095	0.106
IL-12p70	<0.001	1	0.04
IFN-γ	0.297	1	0.163
IL-5	0.06	0.065	0.057
IL-17A	0.012	0.118	0.002
IL-18	0.009	0.026	0.042
MCP-1	0.003	0.011	0.034
IP-10	0.003	0.02	0.008
GRO/KC	0.423	1	0.423
VEGF	0.032	<0.001	0.034
Fractalkine	0.009	0.087	0.053
LIX	0.07	0.02	<0.001
MIP-2	0.105	0.425	<0.001
TNF-α	0.038	0.423	0.003
RANTES	0.005	0.008	<0.001

Syngeneic - Control

Mediator	Skin	Muscle	P. blood
G-CSF	1	0.423	0.747
Eotaxin	1	0.423	0.599
GM-CSF	1	1	0.568
IL-1α	0.07	0.036	0.106
Leptin	0.014	0.151	0.199
MIP-1α	0.016	0.221	0.002
IL-4	0.255	0.007	0.445
IL-1β	0.01	0.428	0.008
IL-2	0.001	<0.001	0.347
IL-6	0.432	0.174	0.27
EGF	0.183	0.032	0.007
IL-13	0.071	0.423	0.115
IL-10	0.351	0.008	0.032
IL-12p70	0.192	<0.001	0.203
IFN-γ	0.074	0.196	0.632
IL-5	0.008	<0.001	0.588
IL-17A	0.098	0.145	0.943
IL-18	0.006	0.002	0.09
MCP-1	<0.001	<0.001	0.026
IP-10	0.061	0.01	0.46
GRO/KC	0.002	0.093	0.146
VEGF	0.082	<0.001	0.235
Fractalkine	0.066	<0.001	0.151
LIX	0.986	0.112	0.559
MIP-2	0.736	0.519	0.036
TNF-α	1	1	0.585
RANTES	0.418	0.006	0.046
